# Identity and timing of protist inoculation affect plant performance largely irrespective of changes in the rhizosphere microbial community

**DOI:** 10.1128/aem.00240-25

**Published:** 2025-03-31

**Authors:** Nathalie Amacker, Zhilei Gao, Alexandre L. C. Jousset, Stefan Geisen, George A. Kowalchuk

**Affiliations:** 1 Ecology and Biodiversity Group, Institute of Environmental Biology, University of Utrecht 98808 , Utrecht, the Netherlands; 2 ECOstyle, Oosterwolde, the Netherlands; 3 Blossom Microbial Technologies BV 525383 , Utrecht, the Netherlands; 4 Laboratory of Nematology, Wageningen University & Research 4508 , Wageningen, the Netherlands; University of Tennessee at Knoxville, Knoxville, Tennessee, USA

**Keywords:** soil, protozoa, bacteria, inoculation time, lettuce

## Abstract

Bacterivorous soil protists can have positive impacts on plant performance, making them attractive targets for novel strategies to improve crop production. However, we generally lack the knowledge required to make informed choices regarding the protist species to be used or the optimal conditions for such amendments. Here, we examined how identity, diversity, and timing of inoculation of well-described protists impacted plant development and rhizosphere microbiome assembly. We first studied the impact of individual inoculation of six well-characterized protists on lettuce growth, with *Cercomonas* sp. S24D2 emerging as the strain with the largest impact on plant growth. In a second step, we created a three-protist species mixture inoculant by adding two protist species (*Acanthamoeba* sp. C13D2 and a heterolobosean isolate S18D10), based on differences in their feeding patterns. We then inoculated *Cercomonas* sp. either individually or in the protist mixture to lettuce plants 1 week before, simultaneously with, or 1 week after seedling transfer. We monitored plant growth and nutrient content, as well as impacts on the resident soil and rhizosphere microbiome composition. We found that early protist inoculation provided the greatest increase in aboveground biomass compared to the non-inoculated control. Single- and mixed-species inoculations had similar impacts on plant development and only minor impacts on prokaryotic community composition. While early inoculation seems to be the most promising timing for eliciting the positive effects of protist amendments, further, more systematic studies will be necessary to determine the conditions and ecological interactions that yield consistent and predictable improvements in plant performance.

**IMPORTANCE:**

The application of microorganisms, including bacterivorous soil protists, has been increasingly suggested as a sustainable agricultural approach. While positive impacts of the presence of predatory protists have been generally reported, the effects of the selected species and amendment conditions are largely unknown. Here, we examined how identity, diversity, and timing of inoculation of well-described protists impacted plant development and rhizosphere microbiome assembly. One species emerged as the one having the strongest impact in our specific system. This result highlights the importance of species selection for optimal outcome, but also suggests a huge potential in the barely investigated protist diversity for targeted application. Furthermore, the application of the inoculants before plant transfer showed the strongest effects on plants, providing some useful and new insights on the optimal time for such amendments.

## INTRODUCTION

Soil predatory protists are abundant and diverse in terrestrial ecosystems (1, 2). They are among the main consumers of bacteria, and their predatory activity can affect plant performance by releasing nutrients from bacterial biomass (3, 4) and by modifying soil bacterial community composition (5, 6). Protist applications have been reported to lead to an increase in the shoot biomass (7, 8), a more elongated and branched root system (9, 10), and a higher concentration of nitrogen (11, 12) and other elements in the shoot (13, 14). Protists can also enhance plant-beneficial bacterial taxa (8, 15). Based on these multiple beneficial effects on plants, protist inoculation may be a promising strategy to support plant growth (16).

Different protist taxa may, however, vary considerably in their food preferences (17, 18), leading to distinct predatory impacts on soil bacterial communities (19, 20). This in turn may differentially impact plants by altering the complex interactions between plants and their associated microbiome (21, 22). Prey preference may be due to specific protist traits such as cell volume and cell flexibility (19, 23), as well as bacterial defense compounds (24), bacterial nutritional values (25, 26), or other bacterial traits (27, 28). We, however, still lack the information necessary to predict the predatory impacts of specific protist isolates based on traits such as morphological (e.g., volume), phylogenetic, physiological (e.g., growth rate), or functional traits (e.g., feeding patterns) (19, 20). Traits may orient a first selection of candidate species, but a subsequent screening step is likely necessary to select the best candidates for a given system (16, 20).

Studies investigating the effects of protist application on plant development have typically focused on one single inoculation time point. However, the success of such amendments is likely to be strongly influenced by the timing of application (29). Significant increases in nutrient turnover may, for instance, only occur after several days due to the time required for the amended protist population to establish and grow sufficiently to exert a significant level of predatory activity (30). More rapid effects have also been reported with observable impact of predatory protists on the bacterial community composition as early as 2 days after inoculation (5). Such impact can, however, also influence bacterial community dynamics over the longer term, as reported by the enrichment of certain bacterial taxa 2–3 weeks after protist inoculation (6). On the other hand, the relative effects of inoculants might also be diminished over time by the increased influence of the plant itself on the rhizosphere community composition (31, 32). If inoculation is too early or too late, the treatment will therefore be less effective. It is therefore important to identify the optimal time of application for increased efficacy of such protist-based treatments.

In addition, the inoculation of multiple protist species could be more effective than single-protist treatments for improving plant performance. In general, the inoculation of multiple protist species is expected to positively affect prey bacterial diversity (33, 34), and higher bacterial diversity has been related to an increase in nitrogen mineralization, which in turn improves plant growth (35). The preferential feeding behavior typically reported for predatory protists (16, 17) may further lead to a higher diversity of plant-beneficial bacteria, and their associated traits, under the predatory pressure of a multispecies protist inoculant (33, 36). Increased protist diversity could thus have a larger and complementary impact on plant performance compared to a single-species inoculation.

In the light of these knowledge gaps, we sought to determine (i) the individual impact of different protist species on plant performance, (ii) the optimal timing of inoculation, and (iii) the impact of single- vs mixed-species inoculation. We first addressed the individual effects of different protist isolates on soil prokaryotic communities and plant performance. To this end, we selected six well-characterized protist isolates covering a range of lineages (Amorphea, TSAR, and “Excavates”) and morphotypes (three amoeboid, two amoebo-flagellates, one flagellate). We studied how their addition to soil influenced prokaryotic community composition, as well as the growth and nutrient content of lettuce plants (*Lactuca sativa*). Based on this first experiment, we selected one protist isolate (*Cercomonas* sp. S24D2) to further test different inoculation time points (i.e., 1 week before transferring plant seedlings, at the time of seedling transfer, and 1 week after seedling transfer) in a greenhouse experiment. In addition, we compared the results of single-protist inoculation to those of a multiple-species inoculant containing a mixture of three protist species (*Cercomonas* sp. S24D2, *Acanthamoeba* sp. C13D2, and a heterolobosean isolate S18D10), chosen to represent a broad range of prey consumption patterns (20). We assessed the effects of our treatments on plant performance by measuring total plant biomass and shoot nutrient content. Changes in the rhizosphere microbial communities were also assessed at the time of harvest, with microbial community analyses targeting prokaryotic and protistan communities via 16S and 18S rRNA gene amplicon sequencing, respectively. We hypothesized that (i) the inoculation of protists would enhance plant performance (such as an increased biomass, a more branched and elongated root system, an increase in nutrient content) and change rhizosphere prokaryotic and protistan community in a species-specific fashion, (ii) an inoculation before seedling transfer would strengthen these effects, (iii) the inoculation of multiple protist species would have a stronger impact on both the plant and the soil microbial community composition compared to single-species additions, and (iv) the impacts on plant properties can be partially explained by changes in the rhizosphere prokaryotic and protistan community.

## MATERIALS AND METHODS

### Protist isolates and preparation

The protists were isolated from a range of environments (sandy soil and growth substrate) in the Netherlands and initially characterized by Gao (23 [Chapter 3, pages 45–62], 37). The protists were isolated from 1 g of soil sample suspended in 20 mL Page’s Amoeba Saline (38) (hereafter referred to as PAS). Soil suspensions were gently shaken for 30 min in a Laboshake (Gerhardt GmbH & Co. KG, Königswinter, Germany), and 1 µL of the mixed soil suspension was pipetted into each well of 96-well plates (Costar, Corning, New York, USA) containing *Escherichia coli* OP50 as food source. After several days of incubation at 15°C, each well was screened to select protists under an inverted Nikon Eclipse TS 100-F microscope (Nikon, Tokyo, Japan). Wells containing potentially pure protist strains were further diluted several times in order to purify a single protist strain. All protist strains were maintained at 15°C and regularly (every 4–6 weeks) transferred to new medium with freshly grown *E. coli*.

For the first experiments (individual inoculation effects), we selected six isolates (*Didymium* sp. P1-1, *Vannella* spp. P33 and P147, *Allovahlkampfia* sp. NL10, *Naegleria* sp. NL81, and *Cercomonas* sp. S24D2) representing some of the main lineages of soil-dwelling, free-living protists (i.e., Amorphea, TSAR, and “Excavates,” according to Burki et al. [39]) and covering various morphotypes. Based on the results of this first experiment, *Cercomonas* sp. S24D2 was further selected to test inoculation time and single- vs mixed-species inoculation in a greenhouse setting. For the mixed-species inoculation, *Cercomonas* sp. was combined with two additional isolates (*Acanthamoeba* sp. C13D2 and a heterolobosean isolate S18D10) chosen because of their different feeding patterns (20).

Before application, each protist isolate was grown separately on *Escherichia coli* OP50 (ca. 10^8^ cells/mL; adjusted to an OD_600_ of 0.5) in PAS from an active stock culture. The day before application, we prepared the protist solution as follows: stock protist cultures were washed three times by gentle centrifugation at 800 × *g* for 5 min (Heraeus Megafuge 40 Centrifuge, Thermo Fisher Scientific, Langenselbold, Germany) to remove spent medium and enrich the protists. Because no visible pellet is formed, we only discarded 75% of the volume before resuspending the cells in the same volume of PAS. Protist density was estimated by transferring three times a volume of 60 or 100 µL in clear polystyrene 96-well microplates with flat bottom (Corning 3370, AZ, USA). Cells were enumerated over three or five screens of the well surface on a monitor connected to an inverted Nikon Eclipse TS 100 microscope (Tokyo, Japan) equipped with a DS Camera Control unit DS-L3 with DS-Fi2 camera head (relay lens: 0.7×) using the 20× objective (final magnification on the monitor: 275×). The solutions were further diluted when necessary to obtain similar final deinnsities (1.3 × 10^4^ ± 1.5 × 10^4^ ind./mL; Table S1). Because these washing steps are not sufficient to fully eliminate all bacterial cells, we quantified the remaining bacteria by serial dilution plating on tryptic soy agar (TSA) (30 g of Bacto tryptic soy broth [TSB], 15 g of agar for 1L; BD, NJ, USA) for 24 h at 28°C (4.6 × 10^6^ ± 7.8 × 10^6^ CFUs/mL; Table S1).

### First experimental setup: testing the effect of individual protist isolates

We studied the individual effects of six protist isolates in a pot experiment set up in a growth chamber with 13 replicates and using lettuce (*Lactuca sativa*) as a model crop. This experiment was performed with a protist-free microbial community to focus on the effects of the specific inoculated isolates.

First, sandy soil was collected from the Utrecht University Botanical Garden (The Netherlands). Six batches of ca. 2 kg of soil were sieved (mesh size: 2 mm) and sterilized via gamma irradiation (>25 kGray; Isotron, Ede, The Netherlands) for use as substrate for the pots. An amount of 75 g was used to obtain a semi-natural protist-free microbial community prepared following an adapted protocol from Rønn et al. (40) and described by Gao (23 [Chapter 3, pages 45–62]). Briefly, 75 g of soil was air-dried, suspended, and mixed in 250 mL 0.1% pyrophosphate buffer (1.68 Na_4_P_2_O_7_ × 10 H_2_O g/L) with a blender (Mix55, BCC Proline, Groupe Fnac Darty, Ivry-sur-Sein, France), and centrifuged at 1,000 × *g* for 40 min at 4°C using a fixed-angle rotor (Thermo Fisher Scientific, Langenselbold, Germany) with lowest acceleration and deceleration to avoid resuspension. The obtained supernatant was first sieved over 50 µm mesh, and then vacuum filtered in a stepwise fashion using autoclaved Whatman glass microfiber filters (Cytiva, Marlborough, USA) from 3 µm, 1.6 µm, to 1.2 µm with a Büchner funnel. A volume of 10 mL of the obtained solution was inoculated in 250 mL CELLSTAR cell culture flask (Greiner Bio-One GmbH, Kremsmünster, Austria) filled with 30 mL of 0.13 mg/L TSB (BD, NJ, USA) supplemented with 100 mg/L agar (Bacto Agar, BD, NJ, USA). After 5 days of incubation at 15°C in the dark, the absence of protists was confirmed by direct observation at 200× and 400× magnification under an inverted Nikon Eclipse TS 100 microscope with phase contrast (Nikon, Tokyo, Japan). By serial dilution plating on 3 g/L TSA plates for 12–24 h at 28°C, we estimated the microbial density to be ca. 3.5 × 10^8^ CFUs/mL. This microbial community was inoculated into the bulk soil and allowed to establish over a period of 11 weeks at room temperature in the dark. Each pot (width: 7 cm, length: 7 cm, height: 8 cm; Lamprecht-Verpackungen GmbH, Göttingen, Germany) was prepared with 200 g of soil and 2 mL of autoclaved demi water, before being distributed over eight different boxes with lids. Each box contained 12 pots. We distributed the 13 replicates of our seven treatments (six different protist isolates and the control for a total number of 91 pots), so that each box had at least one replicate of each treatment. The boxes were used to limit colonization by airborne organisms. A hole of approximately 1 cm depth and 0.5 cm in diameter was made in the middle of each pot, and 1 mL of protist solution was inoculated; the same volume of PAS was used for the non-protist control. Soil moisture was kept constant by matching the daily water consumption with autoclaved water.

Twenty-one days after protist inoculation, lettuce seedlings (*Lactuca sativa* Wonder der Vier Jaargetijden; De Bolster biologische zaden, Epe, The Netherlands) were transferred into each pot. Lettuce seeds were prepared according to an adapted protocol from Trinh et al. (41), including NaOCl-based surface sterilization, a stratification step (3 days at 4°C in the dark), and growth on Murashige and Skoog medium (Minimal Organic Powder Medium, SERVA Electrophoresis GmbH, Heidelberg, Germany) with 3% sucrose (MS3; 8 to 10 seeds per petri dish) in a growth cabinet with a 16 h/8 h day/night regime, at 21°C for 4 days (Phytotron facility, Utrecht University, The Netherlands).

After an additional 21 days of growth, the plants were harvested: the root and shoot were separated from each other. The roots were further washed with demineralized water over a sieve (mesh size: 2 mm) before being placed in Petri dishes containing demineralized water, where they were carefully untangled with tweezers to facilitate image analysis with a WINRhizo scanner (Winrhizo, Regent Instruments Inc, Quebec, Canada). The root surface area was extracted from each image using ImageJ (42). Both the shoot and root were dried at 70°C for 48 h before weight measurement (internal manual and reference 43). Dried shoot material was further processed for inductively coupled plasma atomic emission spectrometry (ICP-AES) and elemental analyzer (EA) analysis (for detailed procedure, see “Plant nutrient measurements,” below).

Soil samples were collected at two different time points (9 days after protist inoculation, before plant seedling transfer, and at the harvest time) for the analysis of the prokaryotic communities via amplicon tag sequencing of the V4 region of the 16S rRNA gene (for detailed procedure, see “Soil microbial community analyses,” below).

### Second experimental setup: testing the effect of time of inoculation and of single- and mixed-species inoculations

This experiment was performed in a greenhouse with non-sterilized soil, following a two-factorial design with three different times of inoculation (7 days before plant transfer, at the time of plant transfer, and 7 days after plant transfer) and two different types of inoculation (single species [*Cercomonas* sp. S24D2] and mixed species [*Cercomonas* sp. S24D2, *Acanthamoeba* sp. C13D2, and heterolobosean isolate S18D10]). Each treatment, including the non-protist treatment used as control, was set in 12 replicates, thereby leading to a total of 84 pots placed following a randomized scheme with an extra layer of plants all around the experimental setup to ensure similar conditions for each plant. Each pot was watered with 10 mL of water on the 1st day, and the moisture was kept constant throughout the experiment via an automatic watering system in use at SoilTech (Biezenmortel, The Netherlands).

Lettuce seeds were prepared as described in the previous section (i.e., NaOCl-based surface sterilization, stratification, growth in controlled conditions 16 h/8 h day/night regime, at 21°C for 4 days) with the modification to use Murashige and Skoog medium without sucrose (MS0; 8 to 10 seeds per Petri dish), as it yielded better germination success. We selected healthy seedlings with a similar phenotype (i.e., ca. 1 cm of main root with abundant root hairs) to be transferred to the pots.

Each pot (external diameter: 13.7 cm, height: 10.8 cm, volume: 0.95 L; pot 14 cm 8° Lw YB, SKU 100.022.000, Van Krimpen B.V., Standdaarbuiten, The Netherlands) was prepared by covering the bottom with a filter (Whatman No. 1, Ø 90 mm, cat no. 1001 090) and 70 mL of wet perlite (35 g ± 1 g), followed by 1,165 g ± 15 g of a sandy soil, prepared as a 50%–50% mixture of river sand and sandy soil at SoilTech (Biezenmortel, The Netherlands); the characteristics of the soil were analyzed by Eurofins Agro Testing Wageningen BV (Wageningen, The Netherlands; Table S2). We also took four soil samples and froze them on the same day at −80°C in Eppendorf tubes for subsequent DNA extractions.

We inoculated the protist solution in the center of each pot, as close as possible to the plants (or future location of the plant), to maximize the chance of the protist to establish close to the rhizosphere, where protist impact is expected to be highest (3, 44). The first application time was performed 1 week before seedling transfer. We created a small hole of ca. 1 cm depth, 0.5 cm in diameter, in the middle of the pot, where the plant would later be transferred, and pipetted 900 µL (single-protist treatment) or 3 × 300 µL (mixture of protists) of the corresponding protist solution (density: 1.3 ± 1.5 × 10^4^ ind./mL; see also Table S1). The three protists were added sequentially to minimize possible interactions between them. A volume of 10 mL of water was added to help protist establishment and to close the hole. The control treatment received 900 µL of PAS. For the second application time, we followed the same procedure but transferred the 5-day-old lettuce seedlings before closing the hole. For the third application time, the protist solution was applied at the base of the plant shoot, as close as possible to the roots.

Plants and rhizosphere soil were harvested after 30 days, which allowed for sufficient aboveground biomass to be formed and limited the spread of an unidentified leaf disease that appeared 48 h before harvest. Most plants (70 from 83; Table S3) had signs of disease; we included the disease incidence in the analyses as the first explanatory variable. During harvest, the shoot was separated from the root, and its fresh weight was recorded before being placed in a paper bag. On the same day, we put the shoot at 70°C to dry. The rhizosphere soil was obtained by perforating with a sterilized metal core the center of each pot to obtain most of the main root and its surrounding soil; this was done to allow reproducible sampling between pots with different root biomass. The obtained samples were stored for 24 h at 4°C before being suspended in a volume of 35 mL of 10 mM MgSO_4_ by horizontal shaking for ca. 10 min (ca. 90 rpm; Gerhardt Schüttelmaschine RO20, Gerhardt GmbH, Bonn, Germany). With sterilized tweezers, we recovered as many roots as possible into a labeled paper bag for weight measurement. The tubes were centrifuged at 3,000 × *g* for 5 min, the supernatant was discarded, and the obtained rhizosphere soil samples were stored at −20°C until further processing to extract DNA.

We further processed the remaining soils to retrieve as many roots as possible. The soil, dried at room temperature, was sieved (mesh diameter: 2 mm) and rinsed with water to eliminate soil and perlite particles. The recovered roots were added to the paper bags with the main root samples previously obtained and dried at 70°C.

Shoot samples were further processed for ICP-AES analysis to obtain Al, Ca, Cd, Cu, Fe, K, Mg, Mn, Na, P, Pb, S, and Zn shoot content (see “Plant nutrient measurements,” below). The shoots and roots were further processed for EA analysis to obtain carbon and nitrogen content (see “Plant nutrient measurements,” below). The root samples did not provide enough material to allow for both analyses, and only carbon and nitrogen content were therefore analyzed. The roots were ground by the use of 2 mL Eppendorf tube containing a metal ball placed on a Retsch MM400 mill (Retsch GmbH, Verder Scientific, Haan, Germany) for 4 min at a frequency of 25 s^−1^. The ground samples were dried at 70°C for 1.5 h. The shoots were first cut into smaller pieces and fitted into bigger metal tubes containing a metal ball and ground similarly using a Retsch MM400 mill (Retsch GmbH, Verder Scientific, Haan, Germany) for 60 s at a frequency of 25 s^−1^. The ground shoots were transferred into 2 mL Eppendorf tubes. The ground shoots and roots were kept in a desiccator until further processing.

### Plant nutrient measurements

The following procedures were used for both experiments.

The procedure for sample preparation for ICP-AES analysis described by Isaac and Johnson (45) was followed with a modification for lower amounts. An amount of ca. 50 mg of dried, ground shoot material was transformed to ash by a 2 h exposition at 500°C in a muffle furnace, digestion in 300 µL of HNO_3_ (65%), complete evaporation on a hot plate at 130°C, and 1 h exposition at 500°C in a muffle furnace. We then added 100 µL HCl (32%) and 9.9 mL deionized water to the ash before processing the samples via the ICP-AES Spectrometer iCAP 6000 Series (Thermo Fisher Scientific, Cambridge, UK) coupled with an AutoSampler ASX-520 (Teledyne CETAC Technologies, NE, USA). The concentration of the following elements was measured: Al, Ca, Cd, Cu, Fe, K, Mg, Mn, Na, P, Pb, S, and Zn. Values were converted from milligram per liter to milligram per plant, based on the dry shoot weight initially measured.

For the measurement of carbon and nitrogen, an amount of 2.5 mg–3.5 mg dry ground shoot or root material was weighed on a Mettler Toledo MX5 microbalance (Mettler Toledo, Greifensee, Switzerland), placed into a small tin cup, and arranged on a 96-well plate as preparation for the elemental analyzer. Calibration standards employed included an empty tin container, 25-(Bis(5-tert-butyl-2-benzo-oxazol-2-yl) thiophene, acetanilide, and atropine. The samples were analyzed via an Interscience EA 1110 CHNS-O elemental analyzer (CE Instruments Ltd, Wigan, England) to obtain the percentage of carbon and nitrogen within the samples. The obtained values were used to calculate the relative and absolute amount of carbon and nitrogen per plant, as well as the C:N ratio.

### Soil microbial community analyses

The following procedure was used for both experiments, except that only the 16S rRNA gene was targeted in the first experiment (testing the individual effect of protist isolates).

DNA was extracted from the original bulk soil before inoculation and rhizosphere soil samples at harvest time by using the DNeasy PowerSoil kit (Qiagen, Hilden, Germany). We followed the manufacturer protocol with slight modifications previously shown to increase yield in our research group: an amount of 0.5 g of soil instead of 0.25 g was taken, and lysis of the cells was obtained using a Retsch mill MM400 (Retsch GmbH, Verder Scientific, Haan, Germany) with Qiagen Tissue Lyser Adapter (Qiagen, Hilden, Germany) fitting up to 24 2 mL Eppendorf tubes at a frequency of 30 s^−1^ for 6 min.

Estimates of total prokaryotic community abundance were obtained via real-time PCR (qPCR) targeting the V4 region of the 16S rRNA gene as follows: extracted environmental DNA was diluted 20 times and prepared for qPCR analysis with a Freedom EVO Tecan robot (Tecan Trading AG, Männedorf, Switzerland). Prokaryotic DNA abundance was measured with an Applied Biosystems ViiA 7 PCR system (Thermo Fisher Scientific, MA, USA) using primers targeting the V4 region of the 16S rRNA gene (341F 5′-CCTACGGGNGGCWGCAG-3′ and 805R 5′-GACTACHVGGGTATCTAATCC-3′; [46, 47]). The qPCR master mix was prepared with solutions of Itaq universal SYBR green supermix (Bio-Rad Laboratories, Veenendaal, The Netherlands), forward primer (5 µM), reverse primer (5 µM), and milliQ water with the respective volume per sample: 5 µL, 0.5 µL, 0.5 µL, and 1.5 µL. A volume of 2.5 µL of template DNA was used per sample. The qPCR was performed by an initial denaturing step at 95°C for 30 s with subsequent cycling for 40 times with a 15 s denaturing step at 95°C and a combined step for annealing and elongation at 60°C for 1 min. Melting curves were obtained based on a standard protocol and used to identify the characteristic peak of the PCR product. Two independent technical replicates were performed for each sample. The commercially available *Escherichia coli* strain B DNA (product number D4889; Sigma Aldrich, Merck, Burlington, MA, USA) was used to make the calibration curve by using seven dilutions from the original known DNA concentration.

To examine the microbial community structure, DNA extracts were used as templates for high-throughput 16S rRNA and 18S rRNA gene amplicon sequencing of prokaryotic and eukaryotic communities, respectively, as carried out by Genome Quebec (Montréal, Canada). Before sending the extracted DNA solution, the obtained DNA yield was assessed on a DeNovix DS-11 Spectrophotometer (DeNovix, Wilmington, DE, USA), and the samples were sent in dry ice to maintain high quality of the DNA. The V4 region of the 16S was sequenced on an Illumina MiSeq sequencing machine (Illumina, CA, USA) using an Illumina MiSeq v3 600 cycle kit and the same set of primers mentioned above (341F and 805R). The V9 region of the 18S was targeted with the primers 1391F and EUkBr (5′-GTACACACCGCCCGTC-3′ and 5′-TGATCCTTCYGCAGGTTCACCTAC-3′ (48, 49); and sequenced on an Illumina MiSeq sequencing machine (Illumina, CA, USA) with the Illumina MiSeq v2 500 cycles kit.

We also investigated the presence of the 18S sequences of the three protist isolates (*Cercomonas* sp. S24D2, *Acanthamoeba* sp. C13D2, and a heterolobosean isolate S18D10; previously obtained [23 {Chapter 3, pages 45–62}, 37]) in the 18S amplicon-sequencing data set. For this purpose, the FASTQs of the 18S amplicon sequences were first converted into FASTA files on the Galaxy server (Galaxy Version 1.1.5+galaxy2 [50]), and then made into a BLAST database (Galaxy Version 2.14.1+galaxy2 [51, 52]). A blastn (megablast) with the query being one of the 18S sequences was then performed against the BLAST database of each treatment (Galaxy Version 2.14.1+galaxy2 [51, 52]). We investigated the presence of sequences with a percentage of identity higher than >95.

The primary analysis of raw FASTQ data was processed using the QIIME2 pipeline (version 2020.6 [53]). The DADA2 workflow (54) was followed with the default settings for error correction, removal of forward and reverse primers, quality filtering, doubleton, and chimera removal of the Illumina amplicon sequences with reads truncated at 200 bp for each single-end read, corresponding to a quality score >30, and allowing forward and reverse sequences to overlap >50 bp. QIIME2’s q2-feature-classifier plugin (55) was used for taxonomy assignment against the SILVA 138 reference database using OTUs at a 99% similarity level from the 515F/806R region of sequences (56) for the prokaryotic community and against the PR2 database (57) for the eukaryotic community. We further processed the data set using version 4.0.3 of the open source statistical software R (58). From the 16S data set, we removed the sequences assigned to chloroplast and mitochondria. To compare prokaryotic communities between the treatments, sequence read numbers were normalized to the minimum sequence number by random subsampling (phyloseq::rarefy_even_depth specifying rngseed(1) for reproducibility of the analysis; Fig. S1a and b for the collector’s curve showing the number of species in function of the sample size). We retained 7,709 amplicon sequence variants (ASVs) for the first experiment and 14,258 ASVs for the second experiment. From the 18S data set of the greenhouse experiment, we focused on the protist community and removed the sequences assigned to Streptophyta, Metazoa, Fungi, and the ones with no assigned Phylum. To compare protistan communities between the treatments, the sequences were normalized to 5,060 reads by random subsampling (phyloseq::rarefy_even_depth specifying rngseed(224) for reproducibility of the analysis). With this threshold, we excluded one sample (single-protist treatment inoculated after plant transfer, S3, replicate 12) with a particularly low library size (2,692). All remaining samples had sufficient depth to approach an asymptote in the collector’s curve (Fig. S1c). After rarefaction, we retained 8,849 ASVs.

### Data analysis

All data analyses were performed using the free, open-access software R, version 4.0.3 (2020-10-10) (58).

### Effect of individual protist isolate

The effect of the protist treatment on the total dry biomass, the shoot dry weight, the root dry weight, and root surface area was investigated with a sequential two-way analysis of variance (ANOVA, type i sum of square), considering first the effect of the box (stats::lm, stats::anova). The ratio between the shoot and root was first log-transformed to address the skewness of the data before the same sequential two-way ANOVA was performed. We analyzed the nitrogen and carbon shoot content both as relative (% per plant) and as absolute amount (milligram per plant). The carbon to nitrogen ratio was calculated based on the absolute values. We performed a sequential two-way ANOVA as described above. Similar to the other plant traits, significant differences in the shoot content of Al, Ca, Cd, Cu, Fe, K, Mg, Mn, Na, P, Pb, S, and Zn in the treatments compared to the control were assessed via a sequential two-way ANOVA as described above. If the ANOVA yielded significant values, a Tukey *post hoc* test was performed to identify which groups were different from each other (stats::TukeyHSD).

We investigated the effect of the protist treatments on the prokaryotic abundance, species richness, and evenness with a sequential two-way ANOVA (type i sum of square) to take into account the box effect (stats::lm and stats::anova) for both time points, followed by a *post hoc* Tukey test in case of significant results (*P* < 0.05; stats::TukeyHSD). The prokaryotic abundance was estimated based on the copy numbers of the 16S gene from our qPCR analysis (59) and adjusted to copy numbers per gram of soil. For the prokaryotic species richness, the three following measures were considered: the observed richness, the Chao1 index, and the Shannon index (phyloseq::plot_richness, [60]). We used Pielou’s evenness, calculated as the Shannon index divided by the logarithm of the observed species richness index on the rarefied data set. We investigated the effect of the protist treatments on the prokaryotic community composition at two time points (prior plant transfer and at the harvest) by performing a sequential two-way permutational multivariate analysis of variance (PERMANOVA; vegan::adonis; Box+protist) on the Bray-Curtis dissimilarity and phylogenetically aware UniFrac and weighted UniFrac distances calculated on the rarefied data set.

In order to obtain a reproducible data analysis, the root of the phylogenetic tree was randomly set with set.seed(224) when using the UniFrac and weighted UniFrac distance. In case of a significant PERMANOVA analysis, we did the pairwise test to identify which treatments were significantly different from each other; multiple testing was controlled by adjusting our *P*-value with the Benjamini and Hochberg correction (61); such correction is indicated as “BH corrected *P*” in the text.

### Effect of time of inoculation and of single- and mixed-species inoculation

We investigated the main effects and potential interaction of our treatments by performing a two-way ANOVA (type I, stats::lm and stats::anova) with first a variable accounting for the health/diseased state of the plant, then the explanatory variables being the time point of application (1: 7 days before plant transfer; 2: simultaneously with plant transfer; 3: 7 days after plant transfer) and the treatment type (single- or mixed-species) (outcome ∼ disease + inoculation time × treatment type) on the following plant properties: fresh shoot weight, dry shoot weight, dry root weight, total biomass, shoot-to-root ratio, shoot carbon content, root carbon content, shoot nitrogen content, root nitrogen content, shoot C:N ratio, root C:N ratio, shoot nutrient content (Al, Ca, Cd, Cu, Fe, K, Mg, Mn, Na, P, Pb, S and Zn). The shoot-to-root ratio was obtained from the ratio of the dry weights. We obtained from the elemental analyzer the relative content of carbon and nitrogen in both shoots and roots and calculated the absolute content in milligram per plant, as well as the C:N ratio on these absolute values. Similarly, we converted the values obtained from the ICP-AES (mg/mL) into absolute shoot content per plant in milligrams for each element. The two-way ANOVA was followed by a *post hoc* Tukey test on the reported significant main effect (*P* < 0.05; stats::aov, stats:TukeyHSD) to identify which treatments were different from each other.

We investigated the presence of the 18S sequence of each protist inoculated in the amplicon-sequencing data set for each treatment with a two-way ANOVA (type I, stats::lm).

Regarding the microbial communities, we investigated the effects of our treatments on the estimated prokaryotic biomass (qPCR) and prokaryotic and protistan alpha diversity (species richness, the Chao1 index, the Shannon index, and the Pielou’s evenness index; phyloseq::plot_richness and phyloseq::estimate_richness) by performing a sequential two-way ANOVA (type I, stats::lm and stats::anova; outcome ∼ disease + inoculation time × treatment type), followed by a *post hoc* Tukey test on the reported significant main effect (*P* < 0.05; stats::aov, stats::TukeyHSD).

The beta diversity between our samples was investigated by using rarefied ASV tables as the basis for the Bray-Curtis dissimilarity and the two phylogenetically aware distances UniFrac and weighted UniFrac. We visualized the different samples based on these distance matrices using phyloseq::ordinate and statistically tested the effect of our treatments by use of a sequential two-way PERMANOVA (vegan::adonis) with first a variable accounting for the health/diseased state of the plant, then with the explanatory variables being the time point of application (7 days before, simultaneously, and 7 days after plant transfer) or the treatment type (single- or mixed-species) (outcome ∼ disease + inoculation time × treatment type). In case of significant results, we further performed pairwise PERMANOVA tests on the significant main effect between the relevant treatments and corrected for multiple testing by using the Benjamini and Hochberg correction (indicated as “BH *P*”; [61]). To enable replication of the analysis, we used set.seed(224) to force R to randomly assign the same root in the phylogenetic tree when computing the UniFrac distances.

Because protist predation may in some cases not affect the whole bacterial community composition (e.g., reference 62), we also investigated differential abundances on the unrarefied 16S and 18S data set with the DESeq R package. Before the analyses, we subdivided our data set into smaller data sets consisting of the treatment of interest and the control and removed rows containing only zeros. A differential expression analysis based on the negative binomial distribution (DESeq; [63]) was used as a basis to visually observe log twofold changes at the genus level compared to the control. Because every sequence had at least one zero occurrence, which makes the calculation of the default logarithmic geometric mean to estimate the size factor impossible, we had to add an arbitrary value of 1 to every count before performing the analysis. In addition, to allow for meaningful visualization without too many ASVs, and as an attempt to control for the reported high false discovery rate (64), we set the alpha value to 0.001 (instead of the default 0.1).

### Relation between effects on the microbial community and plant properties

This analysis only focused on the data from the greenhouse experiment only.

We merged the data of the sequencing data sets per treatment (phyloseq::merge_samples) to calculate the Bray-Curtis dissimilarity and UniFrac/weighted UniFrac distance (phyloseq::distance) relative to the non-protist control as an indicator of the magnitude of induced change on the microbial community composition. We further calculated the averaged difference for each plant property per treatment relative to the non-protist control as an indicator of the magnitude of the effect on the given plant property. We then performed correlation analyses between the magnitude of change induced on the microbial community and on the plant properties (stats::cor.test).

We further explored the potential link between the relative abundance of specific rhizosphere-associated prokaryotic and protist genera and plant properties by running a correlation analysis (stats::cor.test). For this, the relative microbial abundance of the rarefied data sets was used. We corrected the *P*-values for multiple testing using the Bonferroni correction (stats::p.adjust) and visualized the significant correlations (corrected *P* < 0.05) in a heatmap (gplots::heatmap.2).

## RESULTS

### Initial soil bacterial conditions for each setup

Soil samples were analyzed before the addition of any inoculants or plants to assess their initial bacterial community. Both setups had similar initial observed bacterial richness and abundances (first experimental setup: observed species richness: 216 ± 10, averaged bacterial abundance: 6 × 10^8^ ± 1.4 × 10^8^ 16S copies per gram of soil; second experimental setup: observed species richness: 292 ± 28, averaged bacterial abundance: 4.6 × 10^8^ ± 1.5 × 10^8^ 16S copies per gram of soil), but they differed in their composition (Fig. S2). When looking at the relative abundance of the 10 most abundant bacterial phyla, Proteobacteria and Actinobacteria represented more than 75% of the total reads in the first experimental setup. More evenness at the phylum level was observed for the second experimental setup, with, in addition to the Proteobacteria and Actinobacteria, the Chloroflexi and Firmicutes also being highly represented phyla.

### Effect of individual protist isolates

**Fig 1:**
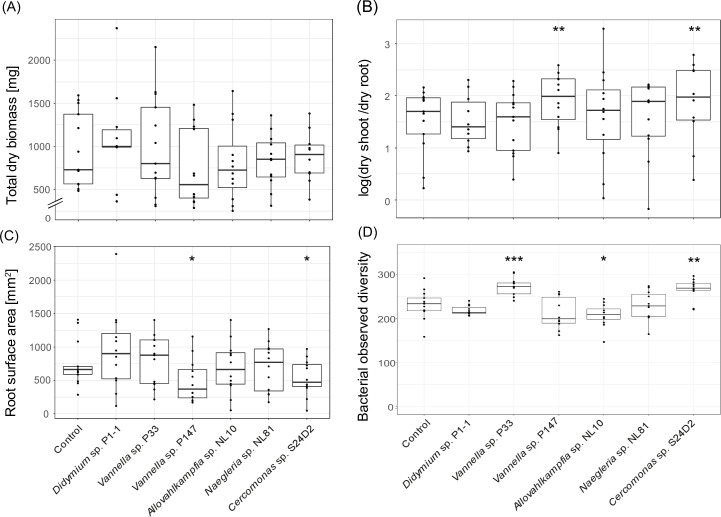
Effect of different protists on various properties of *Lactuca sativa* (A, total dry biomass; B, shoot-to-root ratio [log]; C, root surface area) and on the bacterial alpha diversity at the harvest time (D). Asterisks indicate significant differences compared to control: ***, *P* < 0.001; **, *P* < 0.01; *, *P* < 0.05.

While the total biomass, dry shoot, and root weight were not significantly affected by the protist treatments, we did observe a significant increase in the shoot-to-root ratio in the treatments exposed to *Vannella* sp. P147 [*t*_(67)_ = 3.168, *P* = 0.002] and *Cercomonas* sp. S24D2 [*t*_(67)_ = 3.037, *P* = 0.002] compared to the control (Fig. 1). We also observed a trend toward lower root surface area [*F*_(6,72)_ = 2.084, *P* = 0.066] for the treatments with *Vannella* sp. P147 [*t*_(71)_ = −2.234, *P* = 0.029] and *Cercomonas* sp. S24D2 [*t*_(71)_ = −2.258, *P* = 0.027] compared to the control (Fig. 1). The shoot nutrient content was not significantly different compared to the control for all treatments (Table S4).

Regarding the prokaryotic community structure, we observed the most pronounced effects of the treatments in the rhizosphere soil at the harvest time (Table S5). The treatments with *Vannella* sp. P33 and *Cercomonas* sp. S24D2 induced an increase in the prokaryotic species richness compared to the control, while inoculation with *Allovahlkampfia* sp. NL10 decreased prokaryotic species richness (Fig. 1 and Table S5). While the three indices used to study the beta diversity (Bray-Curtis dissimilarity, unweighted and weighted UniFrac distances) were all found to be significantly affected by the protist treatments (Table S5), only pairwise comparisons based on the Bray-Curtis dissimilarity showed significant differences between treatments. The communities exposed to *Vannella* sp. P33 were significantly different compared to the ones exposed to *Vannella* sp. P147 at both time points [prior to plant transfer: *F*_(1,23)_ = 1.991, BH *P*-value = 0.021; at harvest: *F*_(1,21)_ = 1.713, BH *P*-value = 0.021; Table 1 and Table S5], as well as compared to the control, *Didymium* sp. P1-1, *Allovahlkampfia* sp. NL10, and *Naegleria* sp. NL81 at harvest time (Table 1 and Table S5). We also observed significant differences in the communities exposed to *Cercomonas* sp. S24D2 compared to the control and compared to the treatments with *Didymium* sp. P1-1 and *Allovahlkampfia* sp. NL10 at harvest time (Table 1 and Table S5). An overview of all analyses is provided in Table S5.

**TABLE 1:** Pairwise comparison table between treatments for the Bray-Curtis dissimilarity on the rhizosphere prokaryotic community composition (harvest time)^*a*^

	*Didymium* sp. P1-1	*Vannella* sp. P33	*Vannella* sp. P147	*Allovahlkampfia* sp. NL10	*Naegleria* sp. NL81	*Cercomonas* sp. S24D2
Ctrl	*F*_(1,22)_ = 1.137; *P* = 0.193	***F*_(1,24)_ = 1.778;** **BH *P* = 0.021**	*F*_(1,21)_ = 0.898; *P* = 0.743	*F*_(1,23)_ = 1.202; *P* = 0.087	*F*_(1,22)_ = 0.973; *P* = 0.56	***F*_(1,22)_ = 1.476;** **BH *P* = 0.021**
*Didymium* sp. P1-1	-^*b*^	***F*_(1,22)_ = 1.892;** **BH *P* = 0.021**	*F*_(1,19)_ = 1.035; *P* = 0.394	*F*_(1,21)_ = 1.420; BH *P* = 0.294	*F*_(1,20)_ = 1.264; *P* = 0.042	***F*_(1,20)_ = 1.585;** **BH *P* = 0.042**
*Vannella* sp. P33	-	-	***F*_(1,21)_ = 1.713;** **BH *P* = 0.021**	***F*_(1,23)_ = 2.340;** **BH *P* = 0.021**	***F*_(1,22)_ = 1.868;** **BH *P* = 0.021**	*F*_(1,22)_ = 1.020; *P* = 0.402
*Vannella* sp. P147	-	-	-	*F*_(1,20)_ = 0.999; *P* = 0.486	*F*_(1,19)_ = 1.04; *P* = 0.377	*F*_(1,19)_ = 1.380; BH *P* = 0.126
*Allovahlkampfia* sp. NL10	-	-	-	-	*F*_(1,21)_ = 1.286; BH *P* = 0.546	***F*_(1,21)_ = 1.828;** **BH *P* = 0.021**
*Naegleria* sp. NL81	-	-	-	-	-	*F*_(1,20)_ = 1.388; BH *P* = 0.084

^*a*^ The *P*-values which were adjusted with the Benjamini and Hochberg correction are indicated as “BH *P*.” Significant results (BH *P*-value <0.05) are given in bold.

^*b*^ -, no other significance. To ease readibility and avoid repetition, values are provided only in the upper part of the table.

### Effect of time of inoculation and of single- versus mixed-species inoculation

**Fig 2:**
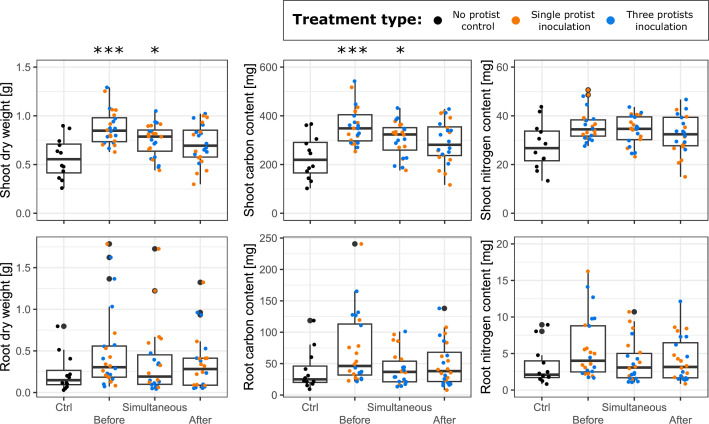
Effect of protist inoculation time (before: 1 week before seedling transplant, together: at time of transplant, after: 1 week after plant transplant) on different plant properties: upper panels, from left to right: shoot dry weight, shoot carbon content, shoot nitrogen content; lower panels, from left to right: root dry weight, root carbon content, root nitrogen content. The treatment type, single or three-species mixture, is given by the colors orange and blue, respectively. Asterisks indicate significant differences for the inoculation time compared to the non-protist control with “*” for *P*-values <0.05 and “***” for *P*-values <0.001.

The disease prevalence was related to an increase in most plant traits including biomass, C and N content, as well as the shoot content in Al, Ca, Cu, K, Mg, Mn, Na, P, S, and Zn; the observed increase related to disease prevalence is probably related to the disease distribution along the treatment, as only one plant from the inoculation time before seedling transfer was exempt of disease signs (Fig. S3; Table S6). The time of inoculation further significantly explained differences in plant aboveground properties, including the shoot fresh and dry weight, carbon content, C:N ratio, and the shoot Ca, Mg, Mn, Na, P, S, and Zn contents. Early inoculation time led to the largest increase in aboveground biomass and nutrient content (Fig. 2 and Table S6 and S7). In contrast to the large effect of inoculation time, the treatment type (single- or mixed-species) only significantly increased the shoot-to-root ratio (*F*_(1,74)_ = 7.093, *P* = 0.009; Table S6) in the three-species mixture (mean ± SD: 5.66 ± 4.92) compared to the single-species inoculation (3.16 ± 2.62) (Table S7). No significant interaction was observed between the two main factors (Table S6).

Sequences corresponding to *Cercomonas* sp. S24D2, *Acanthamoeba* sp. C13D2, and the heterolobosean isolate S18D10 were observed in all treatments including the control (Fig. S4). No significant differences were observed compared to the control for *Cercomonas* sp. S24D2 [*F*_(6,161)_ = 1.136, *P* = 0.344] and the heterolobosean isolate S18D10 [*F*_(3,92)_ = 1.061, *P* = 0.370]. The ANOVA results showed significance for *Acanthamoeba* sp. C13D2 [*F*_(3,92)_ = 2.928, *P* = 0.038], but no significant values were observed in the subsequent Tukey test analysis.

The prevalence of the disease did not have a significant effect on any of the microbial traits investigated [Table 2; for the effect on the bacterial abundance: *F*_(1,74)_ = 0.565, *P* = 0.455]. In line with the first experiment, protist inoculation only had marginal effects on the microbial composition and biodiversity (Table 2). Total prokaryotic abundance, based upon 16S amplicon qPCR, was not significantly affected by any of the treatments [time of inoculation and treatment type; 2.7 × 10^8^ ± 1.3 × 10^8^ 16S copies per gram of soil; *F*_(6,75)_ = 0.311, *P* = 0.929]. Both protist treatments (single- and mixed-species inoculation) reduced prokaryotic alpha diversity, independently of the timing of application (Table 2). Protistan communities were also generally invariable, as we only observed a significant effect of the time of inoculation on protist community composition (Table 2; Fig. S5).

**TABLE 2:** Effect of the treatments on the prokaryotic and protistan community, including the main effects of the inoculation time, the treatment type (single and mixture), and the interaction between both^*b*^

	Microbiome property	Disease		Inoculation time			Treatment type			Interaction		
		*F*-statistic^*a*^	*P*	*F*-statistic^*a*^		*P*	*F*-statistic		*P*	*F*-statistic		*P*
Prokaryotes	16S observed richness	*F*_(1,75)_ = 0.627	0.431	*F*_(3,75)_ = 1.468		0.230	***F*_(1,75)_ = 4.198**		**0.044**	*F*_(2,75)_ = 1.180		0.313
	16S Chao1 index	*F*_(1,75)_ = 0.372	0.544	*F*_(3,75)_ = 1.331		0.271	***F*_(1,75)_ = 4.665**		**0.034**	*F*_(2,75)_ = 1.084		0.343
	16S Shannon	*F*_(1,75)_ = 0.884	0.350	*F*_(3,75)_ = 0.523		0.668	***F*_(1,75)_ = 4.209**		**0.044**	*F*_(2,75)_ = 1.814		0.170
	16S Pielou’s index	*F*_(1,75)_ = 1.195	0.278	*F*_(3,75)_ = 0.253		0.859	*F*_(1,75)_ = 1.269		0.264	*F*_(2,75)_ = 1.267		0.288
	16S Bray-Curtis dissimilarity	*F*_(1,75)_ = 0.880	0.948	*F*_(3,75)_ = 1.023		0.322	*F*_(1,75)_ = 0.983		0.552	*F*_(2,75)_ = 1.013		0.395
	16S UniFrac distance	*F*_(1,75)_ = 1.041	0.142	*F*_(3,75)_ = 0.9954		0.568	*F*_(1,75)_ = 0.974		0.750	*F*_(2,75)_ = 1.035		0.118
	16S weighted UniFrac distance	*F*_(1,75)_ = 0.930	0.559	*F*_(3,75)_ = 1.246		0.062	*F*_(1,75)_ = 1.012		0.397	*F*_(275)_ = 1.228		0.112
Protists	18S observed richness	*F*_(1,73)_ = 0.025	0.875	*F*_(3,73)_ = 0.311		0.818	*F*_(1,73)_ = 0.525		0.471	*F*_(2,73)_ = 0.245		0.783
	18S Chao1 index	*F*_(1,73)_ = 0.043	0.835	*F*_(3,73)_ = 0.273		0.845	*F*_(1,73)_ = 0.423		0.518	*F*_(2,73)_ = 0.236		0.791
	18S Shannon	*F*_(1,73)_ = 0.019	0.892	*F*_(3,73)_ = 1.582		0.201	*F*_(1,73)_ = 2.694		0.105	*F*_(2,73)_ = 0.228		0.797
	18S Pielou’s index	*F*_(1,73)_ = 0.093	0.762	*F*_(3,73)_ = 1.231		0.305	*F*_(1,73)_ = 0.771		0.383	*F*_(2,73)_ = 0.494		0.612
	18S Bray-Curtis dissimilarity	*F*_(1,73)_ = 0.995	0.759	***F*_(3,73)_ = 1.015**		**0.008**	*F*_(1,73)_ = 0.996		0.668	*F*_(2,73)_ = 1.005		0.188
	18S UniFrac distance	*F*_(1,73)_ = 0.940	0.871	*F*_(3,73)_ = 0.998		0.508	*F*_(1,73)_ = 0.968		0.697	*F*_(2,73)_ = 0.997		0.524
	18S weighted UniFrac distance	*F*_(1,73)_ = 1.011	0.337	*F*_(3,73)_ = 0.888		0.661	*F*_(1,73)_ = 1.100		0.438	*F*_(2,73)_ = 1.127		0.375
												

^*a*^ Note that the degree of freedom of the *F*-statistics does not correspond to the theoretical values (inoculation time: *k* − 1 = 3; treatment type: *k* − 1 = 2; *n* − 1 = 83; with *n* the number samples and *k* the number of levels per factor); this is due to the loss of some samples, the use of the same control for both parameter, and the ability of the model to use less degree of freedom.

^*b*^ Significant results are highlighted in bold (*P*-values <0.05). 16S stands for 16S rRNA gene amplicon sequencing, and 18S stands for 18S rRNA gene amplicon sequencing.

**Fig 3:**
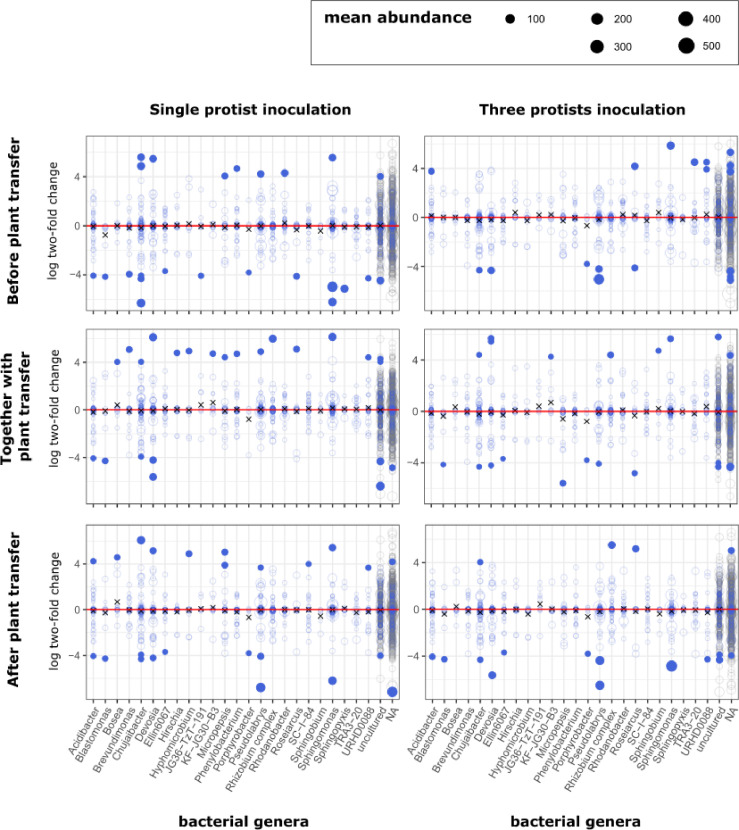
Impact of the protist inoculations (single-species on the left; mixed-species on the right) applied prior (top), simultaneously with (middle), or after (bottom) seedling transfer on bacterial ASVs of the Proteobacteria. Dots above the red line represent bacterial ASVs that were significantly enriched compared to the control, and dots below the red line represent bacterial ASVs that were significantly depleted compared to the control. Open circles are the ASVs showing no significant pattern. Crosses indicate the log twofold average for each given genus, including all non-significantly modified ASVs.

Pairwise differential abundance analysis revealed significant changes in the relative abundance of specific bacterial genera between the different treatments compared to the control (Fig. 3 and Fig. S6 for all phyla). In general, more genera were impacted in the single-protist inoculation (early inoculation: 44 genera, simultaneous inoculation: 29 genera, and late inoculation: 35 genera) compared to the mixed-species inoculation (early inoculation: 27 genera, simultaneous inoculation: 24 genera, and late inoculation: 26 genera). When focusing on the most abundant bacterial phylum observed in our experiment, the Proteobacteria, each treatment displayed distinct patterns of enrichment and depletion for these ASVs compared to the control. For example, ASVs from the genera *Chujaibacter* and *Devosia* were significantly affected by all treatments, but without a consistent direction of the effect. In another example, ASVs from the genus *Bosea* were observed to be two times more abundant in only two of the six treatments compared to the control (single-protist treatment for inoculation time simultaneously and after plant transfer).

In contrast to the bacterial genera, no protist genera were found to be significantly enriched/depleted in any of the treatments compared to the control. This observation includes ASVs that would correspond to the inoculated protist strains.

**Fig 4:**
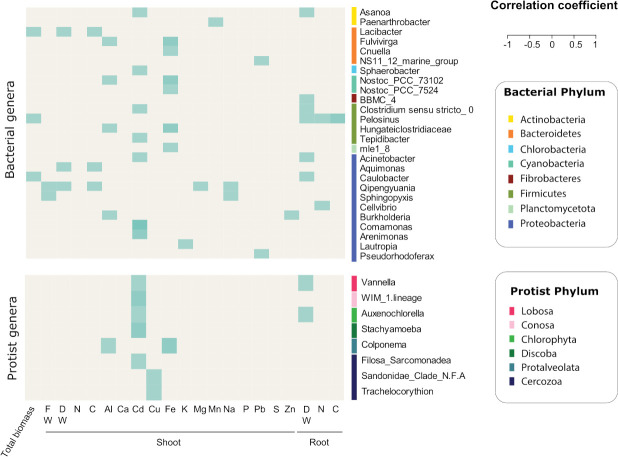
Correlogram between measured plant properties and the relative abundance of bacterial (top) and protist (bottom) genera. FW and DW stand for fresh and dry weight, respectively; the letters stand for the corresponding chemical element. Only correlations with Bonferroni-corrected *P*-values under 0.05 are given.

Due to the limited impact of our treatments on the rhizosphere microbiome composition, we had little discriminatory power to address our fourth hypothesis and investigate potential links between the observed effects on the plant and changes in the microbial community. Nonetheless, we were able to identify potential relations between plant properties and associated microbial communities. The dissimilarity of prokaryotic communities compared to treatments correlated positively with the induced effect on plant growth. In contrast, the impact on plant growth was negatively correlated with the induced change in protistan community composition (Fig. S7). We further found positive correlations between the relative abundances of a total of 25 bacterial genera and several of the measured plant properties. In particular, we observed correlations between cadmium shoot content and seven bacterial genera (*Asanoa* [Actinobacteria], *Sphaerobacter* [Chlorobacteria], *Clostridium* and *Tepidibacter* [Firmicutes], *Acinetobacter*, *Comamonas* and *Arenimonas* [Proteobacteria]), between iron shoot content and five bacterial genera (*Fulvivirga* and *Cnuella* [Bacteroidetes], *Nostoc* [Cyanobacteria], *Hungateiclostridiaceae* [Firmicutes], and the genus “mle” associated with the phylum Planctomycota), as well as between the root dry weight and six bacterial genera (*Asanoa* [Actinobacteria], a genus “BBMC” associated with the phylum Fibrobacteres, *Clostridium* and *Pelosinus* [Firmicutes], *Acinetobacter* and *Caulobacter* [Proteobacteria]) (Fig. 4). When investigating the potential effects of the protist treatment on these bacteria based on the log twofold figures (Fig. 3 and S6), we only found the Proteobacteria *Sphingopyxis* to be reduced in the single-protist treatment inoculated 1 week before plant transfer (log twofold value: −5.117; Fig. 3); *Sphingopyxis* was associated with higher fresh shoot weight and Na shoot content. We also found eight protist genera to be positively correlated with plant properties (Fig. 4). For instance, the cadmium shoot content was correlated with the following five protist genera: *Vannella* (Lobosa), a lineage (WMI-1) part of the phylum Conosa, *Auxenochlorella* (Chlorophyta), *Stachyamoeba* (Discoba), and *Filosa-Sarcomonadea* (Cercozoa). Two other Cercozoa genera (*Sandonidae* and *Trachelochorythion*) were associated with an increase in copper shoot content, and *Colponema* (Protalveolata) was associated with aluminum and iron shoot content.

## DISCUSSION

Protist inoculation of natural soils impacted plant growth and nutrition, with the effects depending on protist isolate identity as well as the timing of inoculation. Inoculation also impacted prokaryotic and protistan community composition, although these effects were rather subtle. The two setups differed in their outcome, which was expected due to some major differences between the two experiments (e.g., soil initial bacterial communities, timing of protist inoculation).

In line with our first hypothesis, the effect of protist inoculation on plant properties and the rhizosphere prokaryotic communities was dependent on the protist identity. The effect on plant properties was, however, relatively small as compared to our greenhouse experiment and to previous studies. Various studies have indeed shown significant direct effects of protist amendment on plant properties, such as increases in shoot biomass and nitrogen content (as reviewed in references 15, 16). The main effect observed in our first experiment was a significant increase in the shoot-to-root ratio for two of the six protist isolates (*Vannella* sp. P147 and *Cercomonas* sp. S24D2). This effect was mostly driven by the trend toward a smaller root surface area rather than an increase in shoot development. Protists have been reported to strongly influence root architecture, though usually toward more elongated and branched root systems (15), without necessarily increasing shoot biomass (9) nor affecting the shoot-to-root ratio (65). Such discrepancies between studies may be partially explained by differences in the protist isolates used. Each species displays its own and distinct collection of traits including volume, growth rate, but also distinct feeding patterns and preferences (19, 20) that influence the outcome as reported in the present study.

With respect to the impact on the prokaryotic communities, amendments with *Cercomonas* sp. S24D2 and *Vannella* sp. P33 increased prokaryotic species richness compared to the control, while *Allovahlkampfia* sp. NL10 decreased prokaryotic community diversity in the rhizosphere. The other protist isolates had no significant impacts on prokaryotic species richness. The decrease of prokaryotic diversity in the treatment with *Allovahlkampfia* sp. NL10 was rather surprising. Indeed, predatory protists are generally expected to feed on the more abundant taxa, providing an advantage to less dominant taxa, thereby leading to higher species richness and evenness (5, 66). Nonetheless, the impact of predatory protists may vary according to the breadth of their feeding niche. For instance, Johnston et al. (67) reported a reduced diversity of the prey community in the presence of a predatory protist described as a generalist. A generalist is expected to feed similarly on all prey and can potentially drive some species to extinction, thus leading to a reduced alpha diversity (67). With respect to the beta diversity, only *Cercomonas* sp. S24D2 and *Vannella* sp. P33 significantly modified the overall community composition at harvest. Such strain-specific impacts of protists on bacterial communities are in line with previous observations, where even closely related protist isolates were shown to have differential impacts on their prey communities (19, 20, 23).

In line with our second hypothesis, we observed that an early inoculation (1 week before seedling transfer) led to the highest increase in plant fresh shoot biomass as compared to the control (1.5×), followed by the simultaneous inoculation (1.3×) and the late inoculation (no significant difference). Such increases in aboveground biomass were mostly consistent with previous studies implementing before (4, 68) or simultaneous protist inoculation with plant transfer (62, 69). Similarly to our study, such increases in the shoot biomass were usually accompanied by an increase in nutrient content (4, 68, 70). Contrary to our hypothesis, the time of inoculation only had very minor impacts on soil microbial community composition, with no significant effects on the overall prokaryotic community structure. One explanation could be the more dominant effect of the lettuce plant itself on the rhizosphere community composition (31), and the impacts of the predators are likely to be dampened over time by the increasingly dominant influence of the plant (32). We did observe an overall shift in the protistan community composition in our late inoculation treatment compared to the control. Interestingly, protistan communities have been suggested to respond more strongly to environmental changes and/or agricultural practices compared to bacterial communities (71, 72).

Contrary to our third hypothesis and to previous studies (62, 68), we did not observe an overall better plant performance in the three-species inoculum treatment compared to the single-species treatment. Berlinches de Gea et al. (73) recently highlighted that biodiversity effects are likely highly context-dependent. These authors reported that the biodiversity effect of protist-based inoculants on plant biomass ranged from being positive under conditions of biotic stress to negative under abiotic stress, with no significant effects under benign conditions. Nonetheless, we did observe a higher shoot-to-root ratio associated with the three-species mixtures compared to the single-protist treatment (1.8×). Shifts toward increased shoot-to-root ratio indicate an alteration in resource allocation toward the production of shoot biomass. Such shifts can be the result of a higher nutrient availability in soil, which may be associated with the activity of protists (11, 69). Also contrary to our hypothesis, the three-species mixture treatments did not show a stronger impact on the soil microbial communities in terms of prokaryotic abundance, richness, evenness, or alpha and beta-diversity. Interestingly, previous studies have reported that the identity of predatory protist species in a mixture is likely critical to the ultimate outcome (74) and that single species can lead to similar effects as observed for mixtures (62). In our study, the *Cercomonas* sp. isolate might have been a more competitive predator compared to the two other predatory protists, thus similarly steering the predatory impact on the prey community in both single- and three-species inoculation (74). It is also worth noting that in our differential abundance analysis, we observed a higher number of bacterial genera being significantly affected in the single-species inoculations compared to the mixed-species inoculations. This observation might stem from emergent, antagonistic multi-predator effects such as competition occurring between the predators in the mixed species, reducing the predatory impact of each individual species (75). Although we did not observe detectable increases in the abundance of the inoculated protist strains, it has been previously shown that successful establishment of microbial inoculants is not necessarily required for the emergence of significant impacts (76). Furthermore, it may be that the population densities of the inoculated strains and their impacts on the resident microbial communities are transient, making their detection dependent on the time of sampling. Early and multiple sampling time points would be helpful for examining these aspects (32).

The modest impacts observed on microbial communities did not provide a large degree of power to address our fourth hypothesis and link changes in the rhizosphere microbial community to changes in plant properties. However, we did observe positive correlations between the UniFrac distances relative to the control for the prokaryotic communities and the magnitude of the effect on plant biomass. Interestingly, and in line with our observation, the highest shoot biomass was previously observed for lettuce plants grown on soil associated with the highest rhizosphere effect (31). In contrast, the UniFrac distance on the protist community composition relative to the control was negatively correlated with the magnitude of effect on plant properties, including total and shoot biomass, and shoot content of some elements (C, Al, Ca, Fe, Mg, Na, and P). This suggests that, while modifications of the prokaryotic community composition might be beneficial for the plant, a stable community composition of the dominant protists might also have positive effects. In particular, the community of predatory protists has been shown to be an important explanatory factor associated with plant health (77, 78). We further identified bacterial and protist genera that were correlated to specific plant properties. Of the bacterial genera identified, some are known to include plant-beneficial microbes, such as *Caulobacter* spp. (79) or *Burkholderia* spp. (80), while others have no known reported plant-beneficial properties (Table S8). Interestingly, *Sphingopyxis* (Proteobacteria), whose population density decreased in the single-protist treatment inoculated 1 week before plant transfer, was previously identified as a good food source for bacterivorous predators (68). We also observed a surprising positive correlation between five protist genera and the shoot content of cadmium, which can be toxic for plants if concentrations are above a certain threshold (81). While protists have been reported to participate in various biogeochemical processes involved in the carbon, nitrogen, phosphorus, magnesium, calcium, and silica cycles (16), to our knowledge, no relation was previously found in relation to cadmium. It should be noted that the analyses described above attempt to link protist-induced changes in microbial community structure to plant properties in an exploratory way, and any observed trends need to be further explored via follow-up experiments targeting these microbial groups.

### Conclusions

We showed that protists have strain-specific impacts on plant properties and soil microbial community composition and that an early protist inoculation had the most positive effect on plant biomass. The biodiversity of introduced protists played a minor role in our experimental system. The application of protists further did not have major, long-lasting effects on the rhizosphere microbial community, although responses of specific taxa were detected. While our study provides some insights into the relations between protist predators, bacterial prey, and plant properties, we encourage an expansion of such approaches to more fully determine the role of multitrophic interactions in plant health and development, as a scientific basis for incorporating protists into strategies for sustainable plant production.

## Data Availability

The data have been deposited in the NCBI BioProject database under accession number PRJNA1192704.

## SUPPLEMENTAL MATERIAL

Supplemental figures aem.00240-25-s0001.docx Figures S1 to S7.

Supplemental tables aem.00240-25-s0002.docx Tables S1 to S8.

ASM does not own the copyrights to Supplemental Material that may be linked to, or accessed through, an article. The authors have granted ASM a non-exclusive, world-wide license to publish the Supplemental Material files. Please contact the corresponding author directly for reuse.
