# Supplementary material for: Identity and timing of protist inoculation affect plant performance largely irrespective of changes in the rhizosphere microbial community
Source: Appl Environ Microbiol. 2025 Mar 31;91(4):e00240-25. doi: 10.1128/aem.00240-25 (PMC12016509; doi:10.1128/aem.00240-25)
Supplement: Supplemental figures — Figures S1 to S7. [file aem.00240-25-s0001.docx]

**Supplementary Figures**


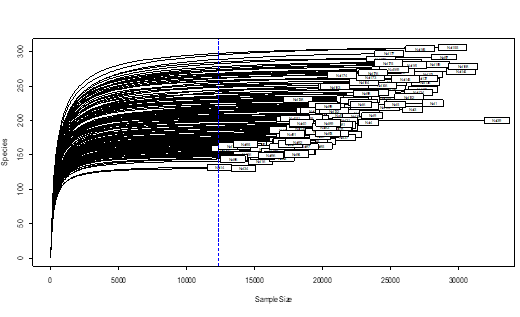

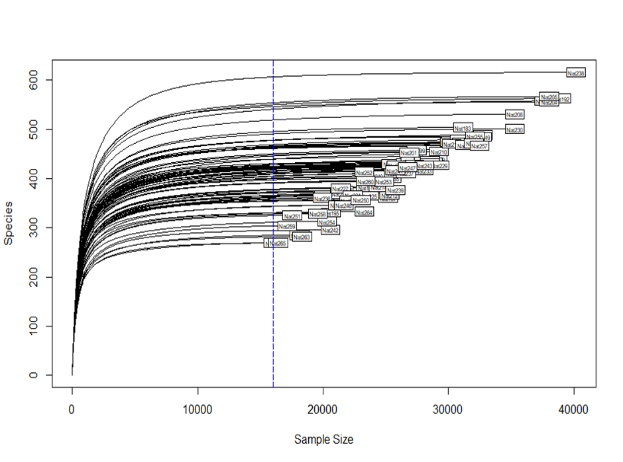

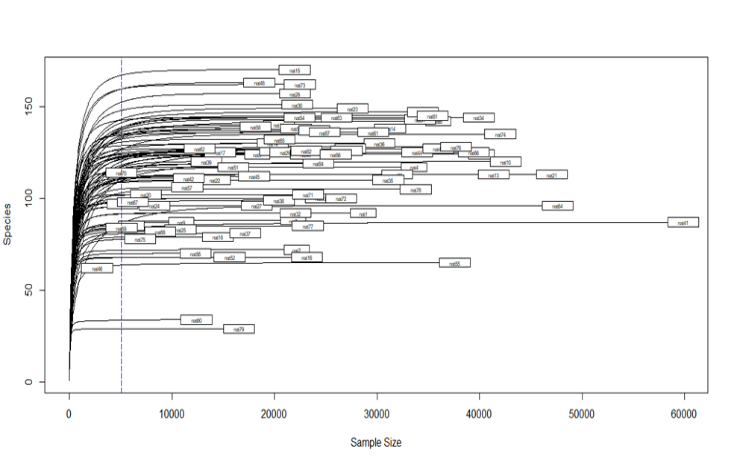


1. Collector’s curve for the 18S amplicon sequencing of the greenhouse experiment; rarefaction was done at 5,060 reads and one sample (S3, replicate 12) was removed.
2. Collector’s curve for the 16S amplicon sequencing of the greenhouse experiment; rarefaction was done at 16,000 reads.
3. Collector’s curve for the 16S amplicon sequencing of the screening experiment; rarefaction was done at 12’4000 reads.

Figure S1: Collector’s curve giving the number of species in function of the sample size. The blue dashed line represents the minimum obtained sample size, which was also used as threshold for the rarefied dataset.

*Figure S2: Distribution of the 10 most abundant bacterial phyla in the initial soil for the first experimental setup (Testing the effect of individual protist isolates, left panel) and for the second experimental setup (Testing the effect of time of inoculation and of single- and mixed-species inoculations, right panel).*

*Figure S3: Distribution of the disease along the different treatments for shoot fresh weight. The left panel shows the shoot fresh weight of the few plants that had no sign of disease. The right panel shows the shoot fresh weight of the plant with sign of the leaf disease. The colors indicate the different treatment type.*


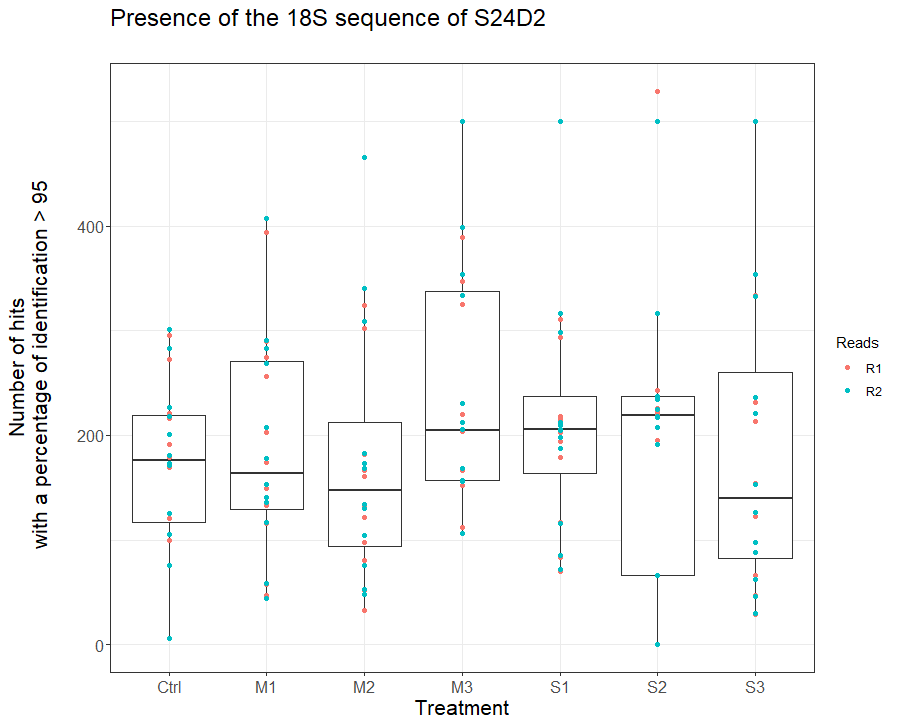


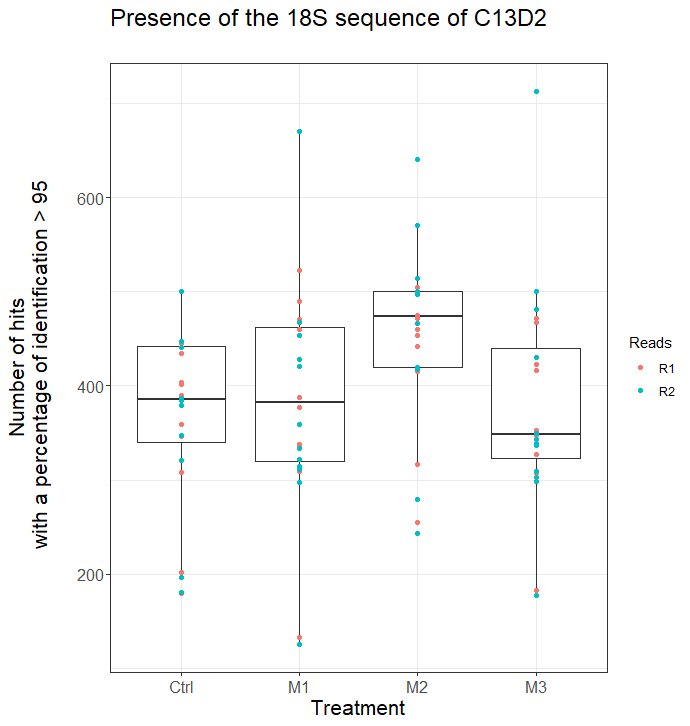

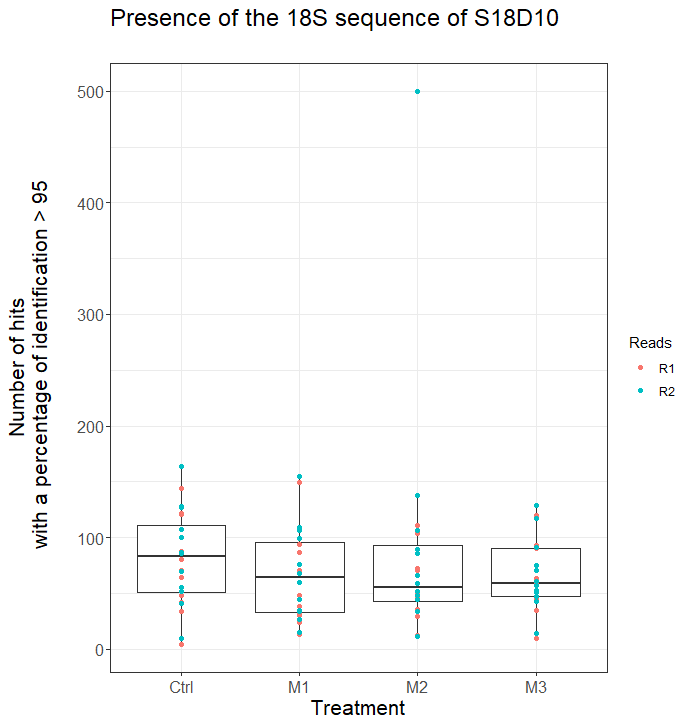


*Figure S4: Number of sequences with a percentage of identity higher than 95% with the 18S sequence of* Cercomonas *sp. S24D2 (upper panel), of* Acanthamoeba *sp. C13D2 (lower left panel) and the heterolobosean isolate S18D10 (lower right panel). The BLAST analysis was performed against both read ends (forward: R1 and reverse:R2).*


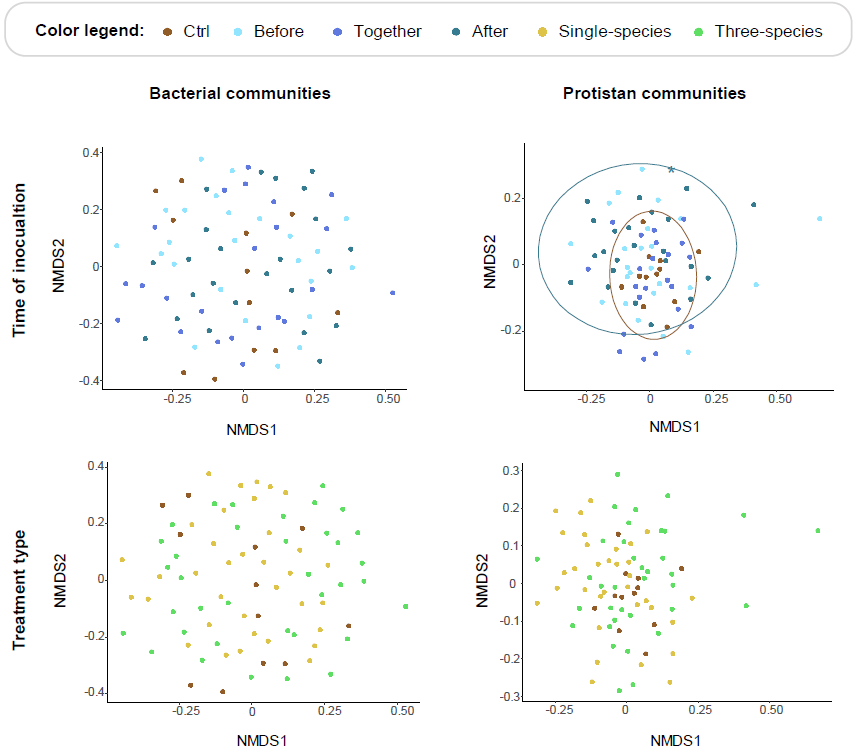


*Figure S5: Effect of the treatments on the bacterial (left panel) and protistan community composition (right panel). The non-metric multidimensional scaling (NMDS) was performed using the Bray-Curtis dissimilarity. The upper panels display the effect of the time of inoculation (one week before seedling transfer, together with seedling transfer, one week after seedling transfer) on the communities and the lower panels display the effects of the treatment type (single- or mixed-species) on the communities. The only significant effect reported was observed between the protistan communities from the late inoculation time (one week after plant transfer) compared to the control; the effect is highlighted with an asterisk and by colored ellipses to help locate the points belonging to the control compared to the ones belonging to the late inoculation time.*


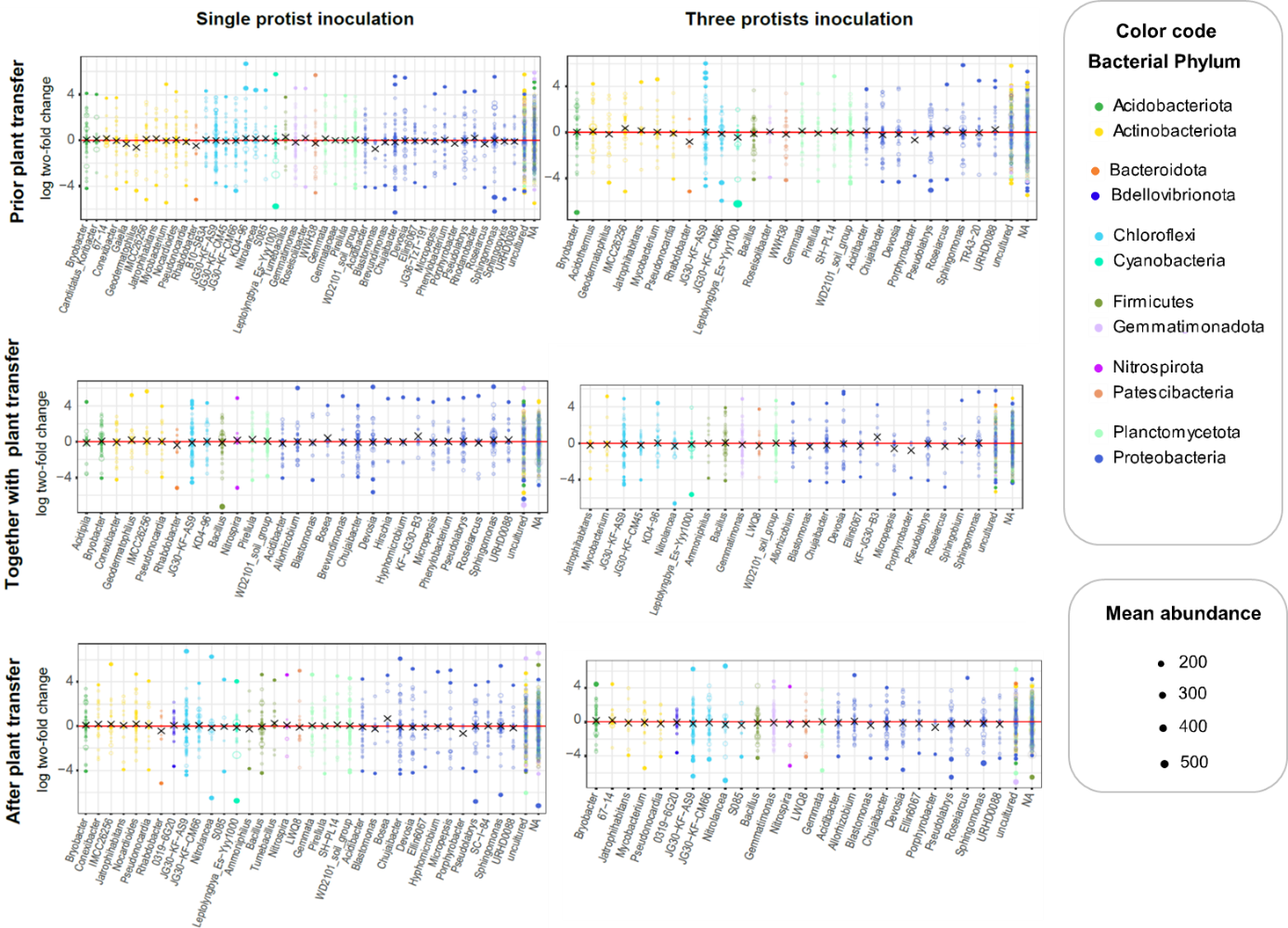


*Figure S6: Impact of the protist inoculations (single-species on the left; mixed-species on the right) applied prior (upper panel), simultaneously with (middle panel) or after (lower panel) seedling transfer on bacterial ASVs. Dots above the red line represent bacterial ASVs that were significantly more abundant in the treatment compared to the control, dots below the red line represent bacterial ASVs that were significantly less abundant in the treatment compared to the control. Open circles are the ASVs showing no significant pattern. Crosses indicate the log two-fold average for each given genus, including all non-significantly modified ASVs.*


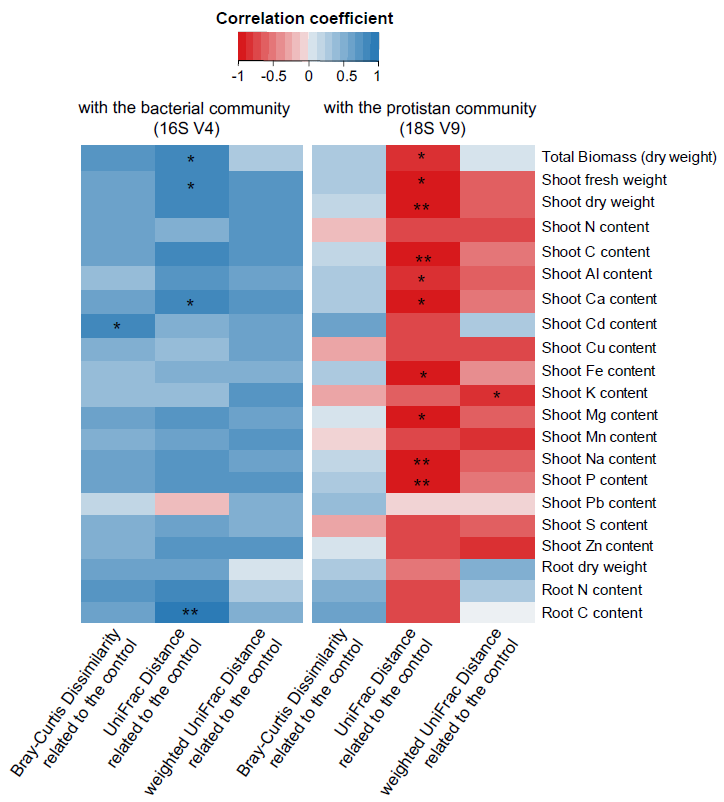


Figure S7: Relation between magnitude of changes on the microbial community composition (bacterial, left; protistan, right) and the magnitude of effect on the different plant properties measured. Asterisks indicate significant correlations with “*” for p < 0.05 and “**” p < 0.01.
