# Supplementary material for: Identity and timing of protist inoculation affect plant performance largely irrespective of changes in the rhizosphere microbial community
Source: Appl Environ Microbiol. 2025 Mar 31;91(4):e00240-25. doi: 10.1128/aem.00240-25 (PMC12016509; doi:10.1128/aem.00240-25)
Supplement: Supplemental tables — Tables S1 to S8. [file aem.00240-25-s0002.docx]

*Table S1: Description of the protist isolates used in the present study. Taxonomic assignment and morphotype are given as described in Gao (2020). The eukaryotic supergroups were assigned according to Burki et al. (2020).*

|  | **Isolate code** | **Eukaryotic supergroup** | **Taxonomic assignment** | **Morpho-type** | **Inoculation time relative to plant transfer[days]** | **Active ind. mL^-1^** | **Cyst mL^-1^** | **CFUs mL^-1^** |
| --- | --- | --- | --- | --- | --- | --- | --- | --- |
| Preliminary screening experiment | P1-1 | Amorphea | *Didymium* sp. | amoeboid | - 21 | 3.05 x 10^4^ | 0 | 1.32 x 10^6^ |
|  | P33 | Amorphea | *Vannella* sp*.* | amoeboid | - 21 | 6.09 x 10^3^ | 0 | 8.65 x 10^5^ |
|  | NL10 | ‘Excavates’ | *Allovahlkampfia* sp*.* | amoebo-flagellate | - 21 | 1.22 x 10^4^ | 0 | 1.45 x 10^6^ |
|  | S24D2 | TSAR* | *Cercomonas* sp*.* | flagellate | - 21 | 6.09 x 10^4^ | 0 | 1.76 x 10^6^ |
|  | P147 | Amorphea | *Vannella* sp*.* | amoeboid | - 21 | 6.09 x 10^3^ | 0 | 1.74 x 10^6^ |
|  | NL81 | ‘Excavates’ | *Naegleria* sp*.* | amoebo-flagellate | - 21 | 3.66 x 10^4^ | 3.66 x 10^4^ | 6.75 x 10^4^ |
| Greenhouse ; single protist | S24D2 | TSAR* | *Cercomonas* sp*.* | flagellate | -7 | 1.08 x 10^4^ | 2.80 x 10^3^ | 1.83 x 10^7^ |
|  |  |  |  |  | 0 | 1.19 x 10^4^ | 1.13 x 10^2^ | 1.02 x 10^6^ |
|  |  |  |  |  | +7 | 1.31 x 10^4^ | 2.49 x 10^2^ | 6.00 x 10^5^ |
| Greenhouse ; mixture of protists | S24D2 | TSAR* | *Cercomonas* sp. | Flagellate | -7 | 9.13 x 10^3^ | 1.03 x 10^4^ | 1.50 x 10^7^ |
|  |  |  |  |  | 0 | 8.59 x 10^3^ | 1.13 x 10^2^ | 1.07 x 10^6^ |
|  |  |  |  |  | +7 | 7.41 x 10^3^ | 1.36 x 10^2^ | 3.67 x 10^5^ |
|  | S18D10 | ‘Excavates’ | Hetero-  lobosea sp. | Amoeboid | -7 | 1.15 x 10^3^ | 3.82 x 10^3^ | 2.83 x 10^7^ |
|  |  |  |  |  | 0 | 2.90 x 10^3^ | 4.07 x 10^3^ | 2.03 x 10^6^ |
|  |  |  |  |  | +7 | 3.75 x 10^3^ | 9.94 x 10^2^ | 2.00 x 10^4^ |
|  | C13D2 | Amorphea | *Acanth-*  *amoeba* sp. | Amoeboid | -7 | 2.03 x 10^3^ | 3.69 x 10^3^ | 6.33 x 10^6^ |
|  |  |  |  |  | 0 | 1.12 x 10^3^ | 3.39 x 10^3^ | 2.00 x 10^6^ |
|  |  |  |  |  | +7 | 2.67 x 10^3^ | 3.30 x 10^3^ | 9.17 x 10^5^ |

** TSAR stands for* ***T****elonemids,* ***S****tramenopiles,* ***A****lveolates, and* ***R****hizaria (Burki et al., 2020); Note that the supergroup ‘Excavates’ is given in quotes as it lacks phylogenetic support (Burki et al., 2020); we still considered it useful to help the reader locate the protist isolates relative to each other and appreciate that, for instance, the* Vannella *spp. are phylogenetically closer to* Didymium *sp. compared to* Naegleria *sp.*

*Table S2: Characteristics of the sandy soil mixture obtained from SoilTech and used in the greenhouse experiment. The analysis was performed in August 2019 by Eurofins Agro Testing Wageningen BV (Wageningen, The Netherlands).*

|  | **mS cm^-1^ at 25°C** | **Cations mmol L^-1^** | | | | | **mmol L^-1^** |
| --- | --- | --- | --- | --- | --- | --- | --- |
| **pH** | **EC** | **NH_4_** | **K** | **Na** | **Ca** | **Mg** | **Si** |
| 5.3 | 0.5 | <0.1 | 0.2 | 0.2 | 1.3 | 0.3 | 0.08 |

| **Anions mmol L^-1^** | | | | | **Trace elements µmol L^-1^** | | | | | |
| --- | --- | --- | --- | --- | --- | --- | --- | --- | --- | --- |
| **NO_3_** | **Cl** | **S** | **HCO_3_** | **P** | **Fe** | **Mn** | **Zn** | **B** | **Cu** | **Mo** |
| 2.4 | 0.2 | 10.3 | < 0.1 | < 0.03 | <0.5 | 13 | 1.8 | 2.8 | 0.4 | <0.1 |

*Table S3 Disease incidence observed at the time of harvest in the greenhouse experiment.*

| **Treatment** | **Diseased plant/total** |
| --- | --- |
| Control | 7/12 |
| Single protist, inoculation time 1 (S1) | 11/12 |
| Mixt protist, inoculation time 1 (M1) | 12/12 |
| Single protist, inoculation time 2 (S2) | 11/12 |
| Mixt protist, inoculation time 2 (M2) | 9/12 |
| Single protist, inoculation time 3 (S3) | 10/12 |
| Mixt protist, inoculation time 3 (M3) | 11/12 |

*Table S4: Results of the sequential two-way ANOVA addressing the main effect of the box and the protist treatments on various plant properties (first experimental setup testing individual effect of protist species). The significant p-values (<0.05) are highlighted in bold.*

|  | **Box** | | **Protist treatment** | |
| --- | --- | --- | --- | --- |
| **Plant property** | **F test** | ***p*** | **F test** | ***p*** |
| Total plant biomass (dry weight, mg) | **F_(7,68)_=5.377** | **< 0.001** | F_(6,68)_=0.842 | 0.542 |
| Shoot biomass (dry weight, mg) | **F_(7,70)_=7.685** | **< 0.001** | F_(6,70)_=0.878 | 0.516 |
| Root biomass (dry weight, mg) | **F_(7,72)_=5.764** | **< 0.001** | F_(6,72)_=1.673 | 0.14 |
| Shoot-to-root ratio (log) | **F_(7,68)_=11.730** | **< 0.001** | **F_(6,68)_=2.466** | **0.032** |
| Root surface area (mm^2^) | **F_(7,72)_=6.50** | **< 0.001** | F_(6,72)_=2.084 | 0.066 |
| Shoot carbon content (%) | **F_(7,70)_=2.316** | **0.035** | F_(6,70)_=0.748 | 0.613 |
| Shoot carbon content (mg) | **F_(7,70)_=10.794** | **< 0.001** | F_(6,70)_=1.601 | 0.16 |
| Shoot nitrogen content (%) | **F_(7,70)_=2.698** | **0.016** | F_(6,70)_=0.487 | 0.816 |
| Shoot nitrogen content (mg) | **F_(7,70)_=14.302** | **< 0.001** | F_(6,70)_=1.161 | 0.337 |
| Shoot C/N ratio | **F_(7,70)_=3.455** | **0.003** | F_(6,70)_=1.694 | 0.135 |
| Al content (mg per plant) | F_(7,63)_=2.064 | 0.061 | F_(6,63)_=0.353 | 0.905 |
| Ca content (mg per plant) | **F_(7,63)_=8.925** | **<0.001** | F_(6,63)_=0.226 | 0.967 |
| Cu content (mg per plant) | **F_(7,63)_= 3.465** | **0.004** | F_(6,63)_=0.083 | 0.998 |
| Cd content (mg per plant) | F_(7,63)_= 1.836 | 0.096 | F_(6,63)_=0.726 | 0.63 |
| Fe content (mg per plant) | F_(7,63)_= 2.153 | 0.051 | F_(6,63)_=0.927 | 0.482 |
| K content (mg per plant) | **F_(7,63)_= 9.405** | **< 0.001** | F_(6,63)_=0.55 | 0.768 |
| Mg content (mg per plant) | **F_(7,63)_= 7.601** | **< 0.001** | F_(6,63)_=0.278 | 0.945 |
| Mn content (mg per plant) | F_(7,63)_= 0.599 | 0.755 | F_(6,63)_=0.215 | 0.971 |
| Na content (mg per plant) | **F_(7,63)_= 6.914** | **< 0.001** | F_(6,63)_= 0.493 | 0.811 |
| P content (mg per plant) | **F_(7,63)_= 7.632** | **< 0.001** | F_(6,63)_= 1.070 | 0.39 |
| Pb content (mg per plant) | F_(7,63)_= 1.643 | 0.14 | F_(6,63)_= 0.677 | 0.669 |
| S content (mg per plant) | **F_(7,63)_= 9.801** | **< 0.001** | F_(6,63)_= 0.382 | 0.888 |
| Zn content (mg per plant) | **F_(7,63)_= 4.928** | **< 0.001** | F_(6,63)_=0.201 | 0.975 |

*Table S5: Results of the sequential two-way ANOVA and PERMANOVA addressing the main effect of the box and the protist treatments on bacterial community structure, prior plant transfer and at harvest time (first experimental setup testing individual effect of protist species). The significant p-values (<0.05) are highlighted in bold.*

|  |  | **Box** | | **Protist treatment** | |
| --- | --- | --- | --- | --- | --- |
| **Time point** | **Bacterial community structure paramter** | **F test** | ***p*** | **F test** | ***p*** |
| Prior plant transfer | Bacterial abundance | **F_(7,75)_=7.777** | **<0.001** | F_(6,75)_=0.264 | 0.952 |
|  | Species richness | F_(7,76)_=1.764 | 0.107 | F_(6,76)_=0.718 | 0.636 |
|  | Chao1 index | F_(7,76)_=1.772 | 0.105 | F_(6,76)_=0.647 | 0.692 |
|  | Shannon index | **F_(7,76)_=19.67** | **<0.001** | F_(6,76)_=0.534 | 0.781 |
|  | Pielou’s evenness | **F_(7,76)_=26.643** | **<0.001** | F_(6,76)_=0.716 | 0.638 |
|  | Bray-Curtis dissimilarity | **F_(7,76)_=1.945** | **0.001** | **F_(6,76)_=1.213** | **0.014** |
|  | Unweighted UniFrac | **F_(7,76)_=2.676** | **0.001** | **F_(6,76)_=1.385** | **0.01** |
|  | Weighted UniFrac | **F_(7,76)_=11.135** | **0.001** | **F_(6,76)_=1.83** | **0.01** |
| At harvest | Bacterial abundance | F_(7,67)_=0.684 | 0.685 | F_(6,67)_=0.716 | 0.638 |
|  | Species richness | **F_(7,67)_=4.345** | **<0.001** | **F_(6,67)_=9.909** | **<0.001** |
|  | Chao1 index | **F_(7,67)_=4.302** | **<0.001** | **F_(6,67)_=10.102** | **<0.001** |
|  | Shannon index | **F_(7,67)_=4.346** | **<0.001** | **F_(6,67)_=5.56** | **<0.001** |
|  | Pielou’s evenness | **F_(7,67)_=4.127** | **<0.001** | F_(6,67)_=2.200 | 0.054 |
|  | Bray-Curtis dissimilarity | **F_(7,67)_=1.411** | **0.001** | **F_(6,67)_=1.425** | **0.001** |
|  | Unweighted UniFrac | **F_(7,67)_= 2.866** | **0.001** | **F_(6,67)_=1.384** | **0.001** |
|  | Weighted UniFrac | **F_(7,67)_=7.006** | **0.001** | **F_(6,67)_=1.874** | **0.004** |

*Table S6: Effect of the disease incidence and of the treatments on plant properties including the main effects of the inoculation time (4 levels: control, before, simultaneously, after), the treatment type (3 levels: control, single and mixture) and the interaction between both. Note that the degree of freedom of the F-statistics do not correspond to the theoretical values (Inoculation time: k-1=3; Treatment type: k-1=2; n-1=83; with n the number samples and k the number of levels per factor); this is due to the loss of some samples, the use of the same control for both parameter and the ability of the model to use less degree of freedom. The* p*-values under 0.05 are highlighted in bold.*

|  | **Disease** | | **Inoculation time** | | **Treatment type** | | **Interaction** | |
| --- | --- | --- | --- | --- | --- | --- | --- | --- |
| **Plant trait** | **F-statistic** | ***p* value** | **F-statistic** | ***p* value** | **F-statistic** | ***p* value** | **F-statistic** | ***p* value** |
| Fresh shoot weight | **F_(1,76)_=38.154** | **< 0.001** | **F_(3,76)_=5.20** | **0.003** | F_(1,76)_=0.65 | 0.422 | F_(2,76)_=0.560 | 0.560 |
| Dry shoot weight | **F_(1,76)_=34.339** | **< 0.001** | **F_(3,76)_=** **4.909** | **0.004** | F_(1,76)_=0.496 | 0.483 | F_(2,76)_=0.420 | 0.658 |
| Dry root weight | **F_(1,74)_=6.147** | **0.015** | F_(3,74)_=0.606 | 0.613 | F_(1,74)_=1.238 | 0.270 | F_(2,74)_=2.040 | 0.137 |
| Total Biomass | **F_(1,74)_=16.909** | **< 0.001** | F_(3,74)_=2.052 | 0.114 | F_(1,74_)=0.375 | 0.542 | F_(2,74)_=1.106 | 0.336 |
| Shoot:Root ratio | F_(1,74)_=0.173 | 0.679 | F_(3,74)_=0.601 | 0.616 | **F_(1,74)_=7.093** | **0.009** | F_(2,74)_=2.025 | 0.139 |
| Shoot Carbon content | **F_(1,76)_=33.419** | **< 0.001** | **F_(3,76)_=4.477** | **0.006** | F_(1,76)_=0.463 | 0.498 | F_(2,76)_=0.273 | 0.762 |
| Shoot Nitrogen content | **F_(1,76)_=30.930** | **< 0.001** | F_(3,76)_=1.888 | 0.139 | F_(1,76)_=0.557 | 0.458 | F_(2,76)_=2.263 | 0.111 |
| Shoot C:N | **F_(1,76)_=20.657** | **< 0.001** | **F_(3,76)_=7.947** | **< 0.001** | F_(1,76)_=0.117 | 0.733 | F_(2,76)_=1.865 | 0.162 |
| Root Carbon content | **F_(1,74)_=4.545** | **0.036** | F_(374)_=1.512 | 0.218 | F_(1,74)_=1.240 | 0.269 | F_(2,74)_=0.422 | 0.657 |
| Root Nitrogen content | **F_(1,74)_=4.904** | **0.030** | F_(3,74)_=0.798 | 0.499 | F_(1,74)_=1.329 | 0.253 | F_(2,74)_=0.699 | 0.500 |
| Root C:N | F_(1,74)_=0.171 | 0.680 | F_(3,74)_=1.738 | 0.167 | F_(1,74)_=0.949 | 0.333 | F_(2,74)_=2.578 | 0.083 |
| Shoot Al | **F_(1,76)_=9.151** | **0.003** | F_(3,76)_=2.495 | 0.066 | F_(1,76)_=0.736 | 0.394 | F_(2,76)_=0.424 | 0.656 |
| Shoot Ca | **F_(1,76)_=51.309** | **< 0.001** | **F_(3,76)_=10.732** | **< 0.001** | F_(1,76)_=0.637 | 0.427 | F_(2,76)_=0.675 | 0.512 |
| Shoot Cd | F_(1,76)_=2.106 | 0.151 | F_(3,76)_=0.385 | 0.764 | F_(1,76)_=0.594 | 0.443 | F_(2,76)_=0.214 | 0.808 |
| Shoot Cu | **F_(1,76)_=17.380** | **< 0.001** | F_(3,76)_=2.361 | 0.078 | F_(1,76)_=1.816 | 0.182 | F_(2,76)_=2.423 | 0.095 |
| Shoot Fe | F_(1,76)_=1.703 | 0.196 | F_(3,76)_=2.371 | 0.077 | F_(1,76)_=3.101 | 0.082 | F_(2,76)_=0.124 | 0.884 |
| Shoot K | **F_(1,76)_=8.313** | **0.005** | F_(3,76)_=1.349 | 0.265 | F_(1,76)_=1.477 | 0.228 | F_(2,76)_=2.321 | 0.105 |
| Shoot Mg | **F_(1,76)_=39.168** | **< 0.001** | **F_(3,76)_=4.581** | **0.005** | F_(1,76)_=0.262 | 0.610 | F_(2,76)_=1.285 | 0.283 |
| Shoot Mn | **F_(1,76)_=53.787** | **< 0.001** | **F_(3,76)_=5.105** | **0.003** | F_(1,76)_=0.970 | 0.328 | F_(2,76)_=3.056 | 0.053 |
| Shoot Na | **F_(1,76)_=32.234** | **< 0.001** | **F_(3,76)_=3.665** | **0.016** | F_(1,76)_=0.622 | 0.433 | F_(2,76)_=0.174 | 0.841 |
| Shoot P | **F_(1,76)_=42.984** | **< 0.001** | **F_(3,76)_=5.491** | **0.002** | F_(1,76)_=3.430 | 0.068 | F_(2,76)_=0.533 | 0.589 |
| Shoot Pb | F_(1,76)_=1.371 | 0.245 | F_(3,76)_=0.285 | 0.836 | F_(1,76)_=2.980 | 0.088 | F_(2,76)_=0.026 | 0.974 |
| Shoot S | **F_(1,76)_=33.462** | **< 0.001** | **F_(3,76)_=3.623** | **0.017** | F_(1,76)_=0.006 | 0.936 | F_(2,76)_=2.565 | 0.084 |
| Shoot Zn | **F_(1,76)_=42.614** | **< 0.001** | **F_(3,76)_=7.304** | **< 0.001** | F_(1,76)_=0.927 | 0.339 | F_(2,76)_=4.639 | 0.013 |

*Table S7: Results of the post hoc Tukey HSD for the significant main effects of either the temporal treatment (before, simultaneous, after) or the treatment type (single species, three-species mixture) according to Table S6.*

| Plant trait | Pairwise comparison | diff | lwr | upr | p adj |
| --- | --- | --- | --- | --- | --- |
| **Fresh shoot weight** | **Before-Ctrl** | **4.810** | **2.197** | **7.424** | **<0.001** |
|  | Simultaneous-Ctrl | 2.608 | -0.006 | 5.221 | 0.051 |
|  | After-Ctrl | 2.344 | -0.269 | 4.958 | 0.095 |
|  | **Simultaneous-Before** | **-2.203** | **-4.337** | **-0.069** | **0.040** |
|  | **After-Before** | **-2.466** | **-4.600** | **-0.332** | **0.017** |
|  | After-Simultaneous | -0.263 | -2.397 | 1.871 | 0.988 |
| **Dry shoot weight** | **Before-Ctrl** | **0.309** | **0.137** | **0.482** | **<0.001** |
|  | **Simultaneous-Ctrl** | **0.178** | **0.006** | **0.351** | **0.040** |
|  | After-Ctrl | 0.146 | -0.026 | 0.319 | 0.125 |
|  | Simultaneous-Before | -0.131 | -0.272 | 0.010 | 0.078 |
|  | **After-Before** | **-0.163** | **-0.304** | **-0.022** | **0.017** |
|  | After-Simultaneous | -0.032 | -0.173 | 0.109 | 0.934 |
| **Shoot : Root ratio** | Single-Ctrl | -1.280 | -4.402 | 1.842 | 0.592 |
|  | Mixt-Ctrl | 1.225 | -1.874 | 4.324 | 0.614 |
|  | **Mixt-Single** | **2.505** | **0.282** | **4.728** | **0.023** |
| **Shoot Carbon content** | **Before-Ctrl** | **129.131** | **55.365** | **202.896** | **<0.001** |
|  | **Simultaneous-Ctrl** | **75.548** | **1.782** | **149.313** | **0.043** |
|  | After-Ctrl | 63.503 | -10.263 | 137.269 | 0.117 |
|  | Simultaneous-Before | -53.583 | -113.812 | 6.646 | 0.099 |
|  | **After-Before** | **-65.628** | **-125.857** | **-5.398** | **0.027** |
|  | After-Simultaneous | -12.045 | -72.274 | 48.185 | 0.953 |
| **Shoot C:N** | **Before-Ctrl** | **1.790** | **0.883** | **2.697** | **<0.001** |
|  | Simultaneous-Ctrl | 0.691 | -0.217 | 1.598 | 0.198 |
|  | After-Ctrl | 0.734 | -0.173 | 1.641 | 0.155 |
|  | **Simultaneous-Before** | **-1.099** | **-1.840** | **-0.359** | **0.001** |
|  | **After-Before** | **-1.056** | **-1.797** | **-0.315** | **0.002** |
|  | After-Simultaneous | 0.043 | -0.698 | 0.784 | 0.999 |
| **Shoot Ca** | **Before-Ctrl** | **9.036** | **5.219** | **12.854** | **<0.001** |
|  | **Simultaneous-Ctrl** | **4.460** | **0.642** | **8.278** | **0.015** |
|  | **After-Ctrl** | **4.060** | **0.242** | **7.878** | **0.033** |
|  | **Simultaneous-Before** | **-4.576** | **-7.694** | **-1.459** | **0.001** |
|  | **After-Before** | **-4.976** | **-8.094** | **-1.859** | **<0.001** |
|  | After-Simultaneous | -0.400 | -3.517 | 2.717 | 0.987 |
| **Shoot Mg** | **Before-Ctrl** | **4.004** | **1.538** | **6.471** | **<0.001** |
|  | Simultaneous-Ctrl | 2.199 | -0.268 | 4.665 | 0.098 |
|  | After-Ctrl | 1.511 | -0.955 | 3.978 | 0.380 |
|  | Simultaneous-Before | -1.806 | -3.819 | 0.208 | 0.095 |
|  | **After-Before** | **-2.493** | **-4.507** | **-0.479** | **0.009** |
|  | After-Simultaneous | -0.687 | -2.701 | 1.326 | 0.807 |
| **Shoot Mn** | **Before-Ctrl** | **0.045** | **0.020** | **0.071** | **<0.001** |
|  | **Simultaneous-Ctrl** | **0.030** | **0.005** | **0.055** | **0.013** |
|  | After-Ctrl | 0.023 | -0.003 | 0.048 | 0.094 |
|  | Simultaneous-Before | -0.015 | -0.036 | 0.006 | 0.226 |
|  | **After-Before** | **-0.022** | **-0.043** | **-0.002** | **0.027** |
|  | After-Simultaneous | -0.007 | -0.028 | 0.013 | 0.788 |
| **Shoot Na** | **Before-Ctrl** | **4.641** | **1.745** | **7.536** | **<0.001** |
|  | **Simultaneous-Ctrl** | **2.975** | **0.079** | **5.870** | **0.042** |
|  | After-Ctrl | 2.218 | -0.677 | 5.114 | 0.193 |
|  | Simultaneous-Before | -1.666 | -4.031 | 0.698 | 0.258 |
|  | **After-Before** | **-2.422** | **-4.787** | **-0.058** | **0.043** |
|  | After-Simultaneous | -0.756 | -3.121 | 1.608 | 0.836 |
| **Shoot P** | **Before-Ctrl** | **3.740** | **1.612** | **5.869** | **<0.001** |
|  | Simultaneous-Ctrl | 1.769 | -0.359 | 3.897 | 0.137 |
|  | After-Ctrl | 1.541 | -0.588 | 3.669 | 0.237 |
|  | **Simultaneous-Before** | **-1.971** | **-3.709** | **-0.233** | **0.020** |
|  | **After-Before** | **-2.200** | **-3.938** | **-0.462** | **0.007** |
|  | After-Simultaneous | -0.228 | -1.966 | 1.509 | 0.986 |
| **Shoot S** | **Before-Ctrl** | **0.160** | **0.053** | **0.267** | **0.001** |
|  | **Simultaneous-Ctrl** | **0.117** | **0.010** | **0.223** | **0.026** |
|  | After-Ctrl | 0.074 | -0.033 | 0.181 | 0.272 |
|  | Simultaneous-Before | -0.043 | -0.130 | 0.044 | 0.562 |
|  | After-Before | -0.086 | -0.173 | 0.001 | 0.054 |
|  | After-Simultaneous | -0.043 | -0.130 | 0.044 | 0.574 |
| **Shoot Zn** | **Before-Ctrl** | **0.033** | **0.017** | **0.050** | **<0.001** |
|  | **Simultaneous-Ctrl** | **0.017** | **0.001** | **0.034** | **0.034** |
|  | **After-Ctrl** | **0.017** | **0** | **0.033** | **0.042** |
|  | **Simultaneous-Before** | **-0.016** | **-0.029** | **-0.002** | **0.014** |
|  | **After-Before** | **-0.016** | **-0.030** | **-0.003** | **0.010** |
|  | After-Simultaneous | -0.001 | -0.014 | 0.013 | 1.000 |

Table S8: Description and/or putative functions of the bacterial and protist genera found to be correlated with plant properties of Lactuca sativa in our greenhouse experiment. Non-exhaustive literature research.

| **Phylum** | **Genus** | **Plant property** | **Reference** | **Description/putative function** |
| --- | --- | --- | --- | --- |
| Actinobacteriota | Asanoa | Cd Shoot; Dry weight root | (Niemhom et al. 2016) | present in bulk and rhizosphere soil |
| Actinobacteriota | Paenarthrobacter | Mn Shoot | (Busse 2016; Riva et al. 2021) | potential plant beneficial activity under drought -> auxin production and degradation of ACC |
| Bacteroidota | Lacibacter | Total Biomass; dry weight shoot; C shoot; | (Yim et al. 2020) | positive correlation between lacibater and root and shoot dry weight |
| Bacteroidota | Fulvivirga | Al Shoot; Fe Shoot | (Jung et al. 2016) | no known relation with plant properties |
| Bacteroidota | Cnuella | Fe Shoot | (Zhao et al. 2014) | isolated from a moss (*Takakia lepidozioides*) |
| Bacteroidota | NS11_12_marine_group | Pb Shoot | (Coclet et al. 2019; Urban et al. 2021) | mainly composed of uncultured bacteria, mainly in marine habitats; without clear ecological implications; Correlated with Pb and Cu |
| Chloroflexi | Sphaerobacter | Cd Shoot | (Hugenholtz and Stackebrandt 2004) | no known relation with plant properties |
| Cyanobacteria | Nostoc_PCC_73102 | Al Shoot; Fe Shoot | (Rai, Söderbäck, and Bergman 2000; Tsai et al. 2012) | N2 -fixing cyanobacteria, plant-symbiosis; N autotrophy; Nostoc has the widest host range from algae to angiosperm; iron in cytochrome |
| Cyanobacteria | Nostoc_PCC_7524 | Fe Shoot |  |  |
| Fibrobacteriota | BBMC_4 | Dry weight root | NA | NA |
| Firmicutes | Clostridium_sensu_stricto_10 | Cd Shoot; Dry weight root | (Gupta and Gao 2009) | cluster I includes many important human and animal pathogens |
| Firmicutes | Pelosinus | Total Biomass; dry weight root; N root; C root | (Shelobolina et al. 2007) | new genus and species, |
| Firmicutes | Hungateiclostridiaceae | Al Shoot; Fe Shoot | (Zhang et al. 2018) | new family; Obligately anaerobic chemoheterotrophs. Most type strains can utilize cellulose as sole carbon and energy source for growth. |
| Firmicutes | Tepidibacter | Cd Shoot | (Slobodkin 2015) | isolated from deep-sea hydrothermal vents. |
| Planctomycetota | mle1_8 | Fe Shoot | NA | NA |
| **Phylum** | **Genus** | **Plant property** | **Ref** | **Comment** |
| Proteobacteria | Acinetobacter | Cd Shoot; Dry weight root | (Brady, Jamal, and Perbin 2021) | found in soil and water, known to colonize intravenous fluids; low virulence, however but capable of causing infection in humans |
| Proteobacteria | Aquimonas | dry weight shoot; C Shoot | (Saha et al. 2005) | new genus ; isolated from a warm spring (India) |
| Proteobacteria | Caulobacter | Total biomass; dry weight root | (Berrios 2022) | plant-growth promoting |
| Proteobacteria | Qipengyuania | Fresh weight shoot; dry weight shoot; C shoot; Mg Shoot; Na Shoot; | (Y. Liu et al. 2021) | One species isolated from Mangrove Soil |
| Proteobacteria | Sphingopyxis | fresh weight shoot; Na shoot | (Sharma et al. 2021) | ability to survive under extreme environments; potential to degrade number of xenobiotics and other environmental contaminants |
| Proteobacteria | Cellvibrio | N root | (Gardner and Keating 2012) | microbial plant cell wall degradation |
| Proteobacteria | Burkholderia_Caballeronia.Paraburkholderia | Al shoot; Zn shoot | (Depoorter et al. 2021; Parke and Gurian-Sherman 2001) | antimicrobial activity ; siderophores; biological control and plant growth promoting; some cause disease in plant too |
| Proteobacteria | Comamonas | Cd Shoot | (Wauters et al. 2003; Liu et al. 2020) | can be human pathogen |
| Proteobacteria | Arenimonas | Cd shoot | (Yuan et al. 2014) | Members of the genus Arenimonas have been isolated from various environments such as seashore sand, rice, an iron mine, soil, compost, fresh water and a eutrophic reservoir. |
| Proteobacteria | Lautropia | K shoot | (Gerner-Smidt et al. 1994) | members can be found in human oral cavities |
| Proteobacteria | Pseudorhodoferax | Pb Shoot | (Bruland et al. 2009) | new genus in 2009; isolated from soil |
|  |  |  |  |  |
| **Phylum** | **Genus** | **Plant property** | **Ref** | **Comment** |
| *Protists* |  |  |  |  |
| Lobosa | Vannella | Cd Shoot; dry weight root | (Maciver, De Obeso Fernandez Del Valle, and Koutsogiannis 2017) | Predator |
| Conosa | WIM_1.lineage_X | Cd Shoot | NA | NA |
| Chlorophyta | Auxenochlorella | Cd Shoot; dry weight root | <https://www.algaebase.org/search/genus/detail/?genus_id=45154> | autotroph |
| Discoba | Stachyamoeba | Cd Shoot | https://www.arcella.nl/stachyamoeba-lipophora/ | amoeboid, found in water and soil |
| Protalveolata | Colponema | Al Shoot; Fe Shoot | (Tikhonenkov et al. 2014) | obligate eukaryovore |
| Cercozoa | Filosa_Sarcomonadea_XXX | Cd Shoot | (Lupatini et al. 2019; Chen et al. 2021) | Abundant in (maize) soil |
| Cercozoa | Sandonidae_Clade_N.F.A | Cu Shoot | (Howe et al. 2011) | no known relation with plant properties |
| Cercozoa | Trachelocorythion | Cu Shoot | <https://www.arcella.nl/trachelocorythion> | shell-bearing |
|  |  |  | (Chatelain et al. 2013) |  |
